# Efficacy and safety of esaxerenone in hypertensive patients with chronic kidney disease, with or without type 2 diabetes mellitus: a pooled analysis of five clinical studies

**DOI:** 10.1038/s41440-025-02259-z

**Published:** 2025-06-30

**Authors:** Haruhito A. Uchida, Jun Wada, Hirohiko Motoki, Koichiro Kuwahara, Kazuomi Kario, Tomohiro Katsuya, Tatsuo Shimosawa, Kenichi Tsujita, Shoko Suzuki, Tomohiro Suedomi, Takashi Taguchi

**Affiliations:** 1 Department of Nephrology, Rheumatology, Endocrinology and Metabolism, Okayama University Faculty of Medicine, Dentistry and Pharmaceutical Sciences, Okayama, Japan; 2 Department of Cardiovascular Medicine, Shinshu University School of Medicine, Nagano, Japan; 3 Division of Cardiovascular Medicine, Department of Medicine, Jichi Medical University School of Medicine, Tochigi, Japan; 4 Katsuya Clinic, Hyogo, Japan; 5 Department of Clinical Laboratory, School of Medicine, International University of Health and Welfare, Chiba, Japan; 6 Department of Cardiovascular Medicine, Graduate School of Medical Sciences, Kumamoto University, Kumamoto, Japan; 7 Data Intelligence Department, Daiichi Sankyo Co., Ltd., Tokyo, Japan; 8 Primary Medical Science Department, Daiichi Sankyo Co., Ltd., Tokyo, Japan

**Keywords:** albuminuria, chronic kidney disease, esaxerenone, morning hypertension, type 2 diabetes mellitus

## Abstract

Effective management of blood pressure (BP) and albuminuria are crucial for suppressing chronic kidney disease (CKD) progression and cardiovascular risks in hypertension. This pooled analysis evaluated the antihypertensive effects, organ-protective effects, and safety of esaxerenone in hypertensive patients with CKD by integrating five clinical studies of esaxerenone. Patients were divided based on type 2 diabetes mellitus (T2DM) status (with or without T2DM) and creatinine-based estimated glomerular filtration rate (eGFR_creat_) (30 to <60 and ≥60 mL/min/1.73 m^2^). Significant changes in morning home BP from baseline at Week 12 were observed in the overall population (mean change −12.8/ − 5.4 mmHg), T2DM subgroups ( − 12.2/ − 4.5 and −14.5/ − 7.8 mmHg), and eGFR_creat_ subgroups ( − 12.5/ − 4.7 and −14.0/ − 6.9 mmHg) (all *P* < 0.001). Bedtime home and office BP showed similar tendencies. Urine albumin-to-creatinine ratio significantly improved from baseline at Week 12 in the overall population (mean change: −55.2%), T2DM subgroups ( − 56.5% and −52.0%), and eGFR_creat_ subgroups ( − 54.6% and −55.4%) (all *P* < 0.001). N-terminal pro-B-type natriuretic peptide levels significantly decreased in the overall population (percent change: −14.1%) and subgroup without T2DM ( − 25.3%). The incidence of serum potassium ≥5.5 mEq/L was lower in the subgroup with T2DM vs without T2DM (3.1% and 11.3%), potentially related to the use of sodium–glucose cotransporter 2 inhibitors. These findings highlight the sustained BP-lowering effect of esaxerenone throughout the day in hypertensive patients with CKD, irrespective of T2DM status, and its significant reduction in albuminuria. The data support the safety and efficacy of esaxerenone in this patient population, underscoring its potential as a valuable therapeutic option.

**Figure:**
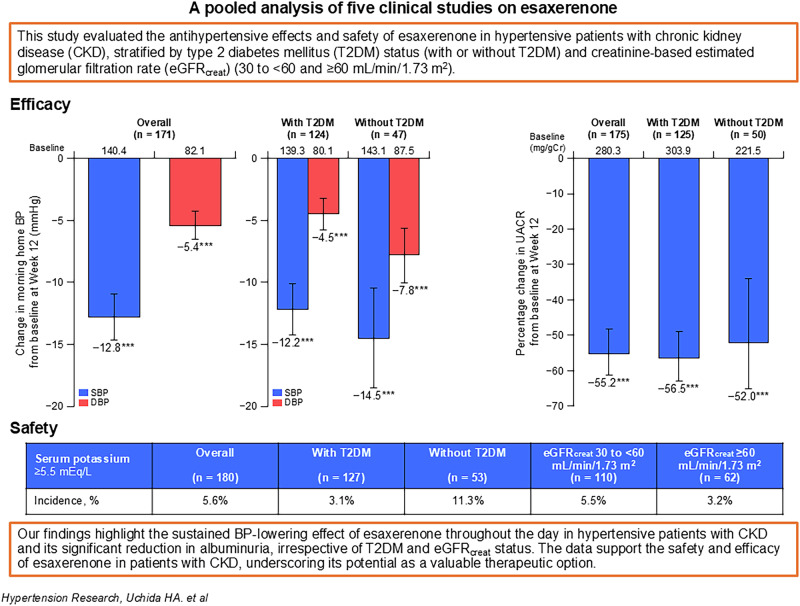
This study showed that esaxerenone significantly lowered morning home, bedtime home, and office BP and UACR in hypertensive patients with CKD, regardless of T2DM status and kidney function (eGFR), and without any novel safety concerns. These highlight the efficacy, organ-protective effects, and safety of esaxerenone in hypertensive patients with CKD.

This study showed that esaxerenone significantly lowered morning home, bedtime home, and office BP and UACR in hypertensive patients with CKD, regardless of T2DM status and kidney function (eGFR), and without any novel safety concerns. These highlight the efficacy, organ-protective effects, and safety of esaxerenone in hypertensive patients with CKD.

## Abstract (web summary)

This study showed that esaxerenone significantly lowered morning home, bedtime home, and office BP and UACR in hypertensive patients with CKD, regardless of T2DM status and kidney function (eGFR), and without any novel safety concerns. These highlight the efficacy, organ-protective effects, and safety of esaxerenone in hypertensive patients with CKD.

## Introduction

Hypertension is a strong risk factor for the development and progression of chronic kidney disease (CKD) [1–4]. CKD itself is a major risk factor for cardiovascular morbidity and mortality, particularly in patients with hypertension [5]. The effective management of blood pressure (BP) and reduction of proteinuria are critical in slowing the progression of CKD and reducing associated cardiovascular risks.

In Japan, the 2019 Japanese Society of Hypertension Guidelines for the Management of Hypertension and the 2023 Japanese Society of Nephrology (JSN) Evidence-based Clinical Practice Guidelines for CKD recommend the use of angiotensin receptor blockers (ARBs)/angiotensin converting enzyme inhibitors (ACEis) as first-line treatment for hypertension in patients with CKD and proteinuria [6, 7]. Despite the availability of these guidelines and various antihypertensive agents, achieving optimal BP control remains a challenge for many patients [8]. A recent study demonstrated that patients with hypertension exhibit highly heterogenous treatment effects and cardiovascular prognoses, with not all benefiting from intensive BP treatment [9], which underscores the importance of treating patients with hypertension according to their background characteristics. Real-world data indicate that calcium channel blockers (CCBs) are the most frequently prescribed medications for hypertensive patients with CKD in Japan, followed by ARBs and ACEis [10].

The 2023 JSN Evidence-based Clinical Practice Guidelines emphasize the importance of reducing albuminuria as a therapeutic target, given its strong association with increased mortality in patients with CKD [7]. Renin–angiotensin system (RAS) inhibitors, mineralocorticoid receptor blockers (MRBs), and sodium–glucose cotransporter 2 inhibitors (SGLT2is) are recommended for the management of albuminuria. Recent large-scale studies have shown that SGLT2is provide favorable cardioprotective and renoprotective effects in patients with CKD, regardless of the presence or absence of type 2 diabetes mellitus (T2DM) [11**–**13], as well as in patients with T2DM alone [14, 15]. Additionally, the MRB finerenone has demonstrated efficacy in suppressing kidney-related adverse events (AEs) in patients with CKD associated with T2DM and has been approved for the treatment of CKD with T2DM, i.e., diabetic nephropathy; however, it is not currently approved for the treatment of hypertension [16**–**19]. This may highlight the need for an MRB with both strong renoprotective and antihypertensive effects.

Esaxerenone, a next-generation non-steroidal MRB, has increased selectivity and potency, a longer half-life, and enhanced bioavailability compared with other MRBs [20, 21]. Esaxerenone has shown favorable BP-lowering effects in hypertensive patients with various characteristics and comorbidities [22**–**26], including patients with albuminuria [27**–**30]. In addition to its BP-lowering effect, previous studies have shown the renoprotective effects of esaxerenone, including albuminuria reduction and remission [27**–**30]. However, the efficacy and safety of esaxerenone in hypertensive patients with albuminuria needs to be examined in a wider range of patients, with or without T2DM, under conditions closer to real-world clinical practice.

The aim of this study was to perform a pooled analysis of five clinical studies to evaluate the efficacy, organ-protective effects, and safety of esaxerenone in hypertensive patients with CKD (defined as a urine albumin-to-creatinine ratio [UACR] ≥30 mg/gCr) according to the presence or absence of comorbid T2DM.

## Methods

### Study design and patients

This study was a pooled subgroup analysis of five clinical studies of esaxerenone: EX-DKD [27], EARLY-NH [24], ESES-LVH [25], ENaK [26], and EAGLE-DH [23]. All studies included were multicenter, prospective, open-label, single-arm trials. Supplementary Table 1 describes the target populations of each study. In all studies, patients received esaxerenone along with basal antihypertensive medications such as ARBs, CCBs, or RAS inhibitors. The details of the eligibility criteria, BP measurements, and biomarker analysis have been previously reported [23**–**27].

This subgroup analysis included hypertensive patients with CKD [23**–**27], defined as albuminuria (UACR ≥ 30 mg/gCr). Patients were divided into two subgroups based on the presence or absence of comorbid T2DM. Patients were further analyzed in a post hoc subgroup analysis based on creatinine-based estimated glomerular filtration rate (eGFR_creat_): 30 to <60 mL/min/1.73 m^2^ and ≥60 mL/min/1.73 m^2^. Among the five studies included, two (EAGLE-DH and ESES-LVH) had 24-week treatment periods; however, only data up to 12 weeks were used in this pooled subgroup analysis.

Ethical approval was obtained from the ethical review committee of the Kitamachi Clinic (Tokyo, Japan), and the study was conducted in accordance with the Declaration of Helsinki and local laws and regulations. The requirement for informed consent was waived because of the secondary use of data from previous studies. This pooled analysis study was registered at the University hospital Medical Information Network Clinical Trials Registry (UMIN): UMIN000054922. Each of the five studies were registered in the Japan Registry of Clinical Trials (jRCT) under the following identifiers: jRCTs061190027 (EX-DKD), jRCTs031200364 (EARLY-NH), jRCTs071190043 (ESES-LVH), jRCTs031210273 (ENaK), and jRCTs031200273 (EAGLE-DH).

### Study endpoints

The efficacy endpoints included the following: time-course change and change from baseline in morning home, bedtime home, and office systolic BP (SBP)/diastolic BP (DBP) at Week 12; proportion of patients who achieved target BP levels; and change and percent change from baseline in UACR and N-terminal prohormone of brain natriuretic peptide (NT-proBNP) at Week 12. Two criteria were used to define target BP levels in accordance with the Japanese Society of Hypertension 2019 Guidelines (criterion 1: home BP < 135/85 mmHg, office BP < 140/90 mmHg; and criterion 2: home BP < 125/75 mmHg, office BP < 130/80 mmHg for patients aged <75 years, those with CKD [UACR ≥ 30 mg/gCr], or those with diabetes mellitus) [6].

The safety endpoints included the following: treatment-emergent AEs (TEAEs) and adverse drug reactions (ADRs); change from baseline and time-course change in eGFR_creat_ and serum potassium (K) levels; and proportion of patients with serum K ≥ 5.5 mEq/L within 12 weeks after study drug administration.

The exploratory endpoints were the following: proportion of patients with improved UACR; proportion of patients with a ≥ 30% reduction in UACR from baseline; and proportion of patients with UACR remission. Improved UACR was defined as an improvement in UACR category at Week 12 in patients with baseline UACR A2 or A3 categories. Remission was defined as the transition to the UACR A1 category (UACR < 30 mg/gCr) combined with a ≥ 30% reduction in UACR from baseline. Patients were categorized based on their UACR levels as follows: those with a UACR < 30 mg/gCr were included in the A1 subcohort, those with a UACR of 30 to <300 mg/gCr were included in the A2 subcohort, and those with a UACR of 300 to <1000 mg/gCr were included in the A3 subcohort.

### Statistical analysis

No sample size calculations were conducted because this was a pooled analysis of existing trial data. The full analysis set (FAS) of each study was used to evaluate the efficacy endpoints, the per-protocol set (PPS) was used for the sensitivity analysis, and the safety analysis set of each study was used to evaluate the safety endpoints. The definitions for each analysis set have been previously reported [23**–**27].

For the difference in BP measurements between baseline and Week 12, point estimates and 95% confidence intervals (CIs) were calculated, and comparisons were made using paired *t*-tests. The change and percent change from baseline in UACR and NT-proBNP were evaluated using similar significance tests. For the proportion of patients who achieved target BP levels, 95% CIs were calculated using the Clopper–Pearson method. Missing 12-week data were not imputed in this study. TEAEs and ADRs were coded by System Organ Class and Preferred Term according to the Medical Dictionary for Regulatory Activities, version 27.0.

Statistical significance was set at 5% (two-sided). All statistical analyses were performed using SAS version 9.4 (SAS Institute Inc., Cary, NC, USA).

## Results

### Patients

The total numbers of patients in the safety analysis set and FAS of the five esaxerenone studies were 493 and 479, respectively. Among them, 180 (with T2DM, 127; without T2DM, 53) hypertensive patients with CKD were included in the safety analysis set; 175 (with T2DM, 125; without T2DM, 50) were included in the FAS; and 145 (with T2DM, 102; without T2DM, 43) were included in the PPS.

The background characteristics of patients in the FAS are summarized in Table 1. The proportion of male patients was higher in the subgroup with T2DM vs the subgroup without T2DM (64.8% vs 48.0%). The proportion of patients aged ≥65 years was higher in the subgroup with T2DM vs the subgroup without T2DM (70.4% vs 56.0%). The proportion of patients with body mass index ≥25 kg/m^2^ was also higher in the subgroup with T2DM vs the subgroup without T2DM (64.0% vs 46.0%). Mean morning home SBP/DBP was 139.3/80.1 and 143.1/87.5 mmHg in the subgroups with and without T2DM, respectively. The mean ± standard deviation (SD) eGFR_creat_ was lower in the subgroup with T2DM vs the subgroup without T2DM (57.3 ± 16.4 vs 69.3 ± 20.6 mL/min/1.73 m^2^). Mean ± SD serum K levels were similar in both subgroups (4.2 ± 0.4 and 4.1 ± 0.5 mEq/L, respectively). The final dose of esaxerenone was 1.25 mg in 34.4% and 16.0%; 2.5 mg in 41.6% and 52.0%; and 5 mg in 24.0% and 32.0% of patients in the subgroups with and without T2DM, respectively. The distribution of patients using basal antihypertensive drugs in the overall population was as follows: 34.3% for RAS inhibitors, 26.3% for CCBs, and 39.4% for both drug classes. Similar results were obtained in the PPS (Supplementary Table 2).

**Table 1:** Patient characteristics (FAS)

	Overall	With T2DM	Without T2DM	eGFR_creat_ 30 to < 60 mL/min/1.73 m^2^	eGFR_creat_ ≥ 60 mL/min/1.73 m^2^
	(N = 175)	(n = 125)	(n = 50)	(n = 108)	(n = 59)
Sex, male	105 (60.0)	81 (64.8)	24 (48.0)	68 (63.0)	34 (57.6)
Age, years	67.6 ± 11.3	68.1 ± 10.3	66.4 ± 13.5	71.7 ± 8.7	60.9 ± 12.14
≥65	116 (66.3)	88 (70.4)	28 (56.0)	90 (83.3)	24 (40.7)
Body mass index, kg/m^2^	26.2 ± 4.4 n = 174	26.6 ± 3.8 n = 124	25.3 ± 5.4 n = 50	26.1 ± 3.6 n = 107	26.9 ± 5.5 n = 59
≥25	103 (58.9)	80 (64.0)	23 (46.0)	66 (61.1)	36 (61.0)
Current smoker	40 (22.9)	27 (21.6)	13 (26.0)	21 (19.4)	18 (30.5)
Alcohol use	73 (41.7)	49 (39.2)	24 (48.0)	41 (38.0)	29 (49.2)
Comorbidities					
T2DM	125 (71.4)	125 (100.0)	0 (0.0)	91 (84.3)	33 (55.9)
Dyslipidemia	123 (70.3)	90 (72.0)	33 (66.0)	77 (71.3)	39 (66.1)
Hyperuricemia	70 (40.0)	44 (35.2)	26 (52.0)	43 (39.8)	20 (33.9)
Heart failure	33 (18.9)	16 (12.8)	17 (34.0)	12 (11.1)	14 (23.7)
Initial dose of esaxerenone					
1.25 mg	119 (68.0)	101 (80.8)	18 (36.0)	104 (96.3)	13 (22.0)
2.5 mg	56 (32.0)	24 (19.2)	32 (64.0)	4 (3.7)	46 (78.0)
Final dose of esaxerenone					
1.25 mg	51 (29.1)	43 (34.4)	8 (16.0)	46 (42.6)	3 (5.1)
2.5 mg	78 (44.6)	52 (41.6)	26 (52.0)	40 (37.0)	34 (57.6)
5 mg	46 (26.3)	30 (24.0)	16 (32.0)	22 (20.4)	22 (37.3)
Hypertension disease duration, years	10.1 ± 9.0 n = 127	10.9 ± 8.8 n = 90	8.1 ± 9.5 n = 37	11.1 ± 9.9 n = 76	8.7 ± 7.5 n = 47
Morning home SBP, mmHg	140.4 ± 12.7 n = 171	139.3 ± 12.7 n = 124	143.1 ± 12.4 n = 47	139.5 ± 12.2 n = 104	141.7 ± 14.1 n = 59
Morning home DBP, mmHg	82.1 ± 11.7 n = 171	80.1 ± 11.2 n = 124	87.5 ± 11.3 n = 47	78.4 ± 11.1 n = 104	88.6 ± 10.4 n = 59
Bedtime home SBP, mmHg	134.5 ± 14.0 n = 172	133.9 ± 14.5 n = 124	136.2 ± 12.5 n = 48	134.1 ± 14.2 n = 106	134.8 ± 14.0 n = 58
Bedtime home DBP, mmHg	77.2 ± 11.7 n = 172	75.7 ± 12.0 n = 124	81.0 ± 9.9 n = 48	74.0 ± 11.8 n = 106	82.9 ± 9.4 n = 58
Office SBP, mmHg	145.6 ± 15.3	143.4 ± 13.7	150.9 ± 17.7	145.7 ± 13.9	145.0 ± 17.7
Office DBP, mmHg	82.3 ± 11.6	79.9 ± 10.4	88.2 ± 12.6	79.0 ± 10.8	88.0 ± 11.5
Basal antihypertensive agents					
RAS inhibitor	60 (34.3)	39 (31.2)	21 (42.0)	30 (27.8)	26 (44.1)
CCB	46 (26.3)	17 (13.6)	29 (58.0)	17 (15.7)	25 (42.4)
Both RAS inhibitor and CCB	69 (39.4)	69 (55.2)	0 (0.0)	61 (56.5)	8 (13.6)
Diabetes medications					
SGLT2i	66 (37.7)	66 (52.8)	0 (0.0)	49 (45.4)	17 (28.8)
Biguanide	61 (34.9)	61 (48.8)	0 (0.0)	41 (38.0)	20 (33.9)
Thiazolidinedione	8 (4.6)	8 (6.4)	0 (0.0)	6 (5.6)	2 (3.4)
Sulfonylurea	21 (12.0)	21 (16.8)	0 (0.0)	20 (18.5)	1 (1.7)
Glinide	8 (4.6)	8 (6.4)	0 (0.0)	7 (6.5)	1 (1.7)
DPP-4 inhibitor	70 (40.0)	70 (56.0)	0 (0.0)	52 (48.1)	18 (30.5)
Alpha glucosidase inhibitor	15 (8.6)	15 (12.0)	0 (0.0)	14 (13.0)	1 (1.7)
Insulin	12 (6.9)	12 (9.6)	0 (0.0)	10 (9.3)	2 (3.4)
GLP1 agonist	5 (2.9)	5 (4.0)	0 (0.0)	4 (3.7)	1 (1.7)
Number of diabetes medications					
1	28 (16.0)	28 (22.4)	0 (0.0)	22 (20.4)	6 (10.2)
2	35 (20.0)	35 (28.0)	0 (0.0)	26 (24.1)	9 (15.3)
3	50 (28.6)	50 (40.0)	0 (0.0)	37 (34.3)	13 (22.0)
None	62 (35.4)	12 (9.6)	50 (100.0)	23 (21.3)	31 (52.5)
NT-proBNP, pg/mL	133.3 ± 199.3 80.0 (37.0, 130.0) n = 170	123.2 ± 184.0 80.0 (37.5, 129.5) n = 120	157.5 ± 232.0 75.7 (37.0, 162.0) n = 50	159.2 ± 228.8 92.0 (50.0, 154.5) n = 104	85.3 ± 111.7 43.0 (31.0, 82.0) n = 58
<125	123 (70.3)	88 (70.4)	35 (70.0)	69 (63.9)	48 (81.4)
≥125	47 (26.9)	32 (25.6)	15 (30.0)	35 (32.4)	10 (16.9)
UACR, mg/gCr	280.3 ± 536.0 113.6 (47.9, 304.0)	303.9 ± 578.7 150.2 (60.4, 332.9)	221.5 ± 409.4 69.9 (39.9, 173.2)	369.7 ± 652.1 177.1 (71.0, 472.2)	127.1 ± 174.9 57.9 (37.9, 139.0)
≥30	175 (100.0)	125 (100.0)	50 (100.0)	108 (100.0)	59 (100.0)
Serum potassium, mEq/L	4.2 ± 0.4 n = 167	4.2 ± 0.4 n = 124	4.1 ± 0.5 n = 43	4.3 ± 0.4 n = 108	4.1 ± 0.5 n = 59
<4.5	125 (71.4)	93 (74.4)	32 (64.0)	77 (71.3)	48 (81.4)
≥4.5	42 (24.0)	31 (24.8)	11 (22.0)	31 (28.7)	11 (18.6)
eGFR_creat_, mL/min/1.73 m^2^	60.4 ± 18.3 n = 167	57.3 ± 16.4 n = 124	69.3 ± 20.6 n = 43	49.7 ± 7.7 n = 108	80.0 ± 15.5 n = 59
30 to <60	108 (61.7)	91 (72.8)	17 (34.0)	108 (100.0)	0 (0.0)
≥60	59 (33.7)	33 (26.4)	26 (52.0)	0 (0.0)	59 (100.0)
Plasma aldosterone, pg/mL	54.1 ± 37.9 n = 157	59.9 ± 39.9 n = 117	36.9 ± 24.5 n = 40	58.9 ± 33.7 n = 103	47.4 ± 46.8 n = 46
<120	151 (86.3)	112 (89.6)	39 (78.0)	99 (91.7)	44 (74.6)
≥120	6 (3.4)	5 (4.0)	1 (2.0)	4 (3.7)	2 (3.4)
Plasma renin activity, ng/mL/h	3.0 ± 4.4 n = 164	3.5 ± 4.9 n = 118	1.7 ± 1.9 n = 46	2.9 ± 3.2 n = 104	3.4 ± 6.3 n = 52
<1.0	56 (32.0)	33 (26.4)	23 (46.0)	31 (28.7)	22 (37.3)
≥1.0	108 (61.7)	85 (68.0)	23 (46.0)	73 (67.6)	30 (50.8)

Data are n (%), mean ± standard deviation, or median (Q1, Q3)

*CCB* calcium channel blocker, *DBP* diastolic blood pressure, *DPP-4* dipeptidyl peptidase-4, *eGFR*_creat_ creatinine-based estimated glomerular filtration rate, *FAS* full analysis set, *GLP1* glucagon-like peptide-1, *NT-proBNP* N-terminal prohormone of brain natriuretic peptide, *Q* quartile, *RAS* renin–angiotensin system, *SBP* systolic blood pressure, *SGLT2i* sodium–glucose cotransporter 2 inhibitor, *T2DM* type 2 diabetes mellitus, *UACR* urine albumin-to-creatinine ratio

### Antihypertensive effects

**Fig. 1:**
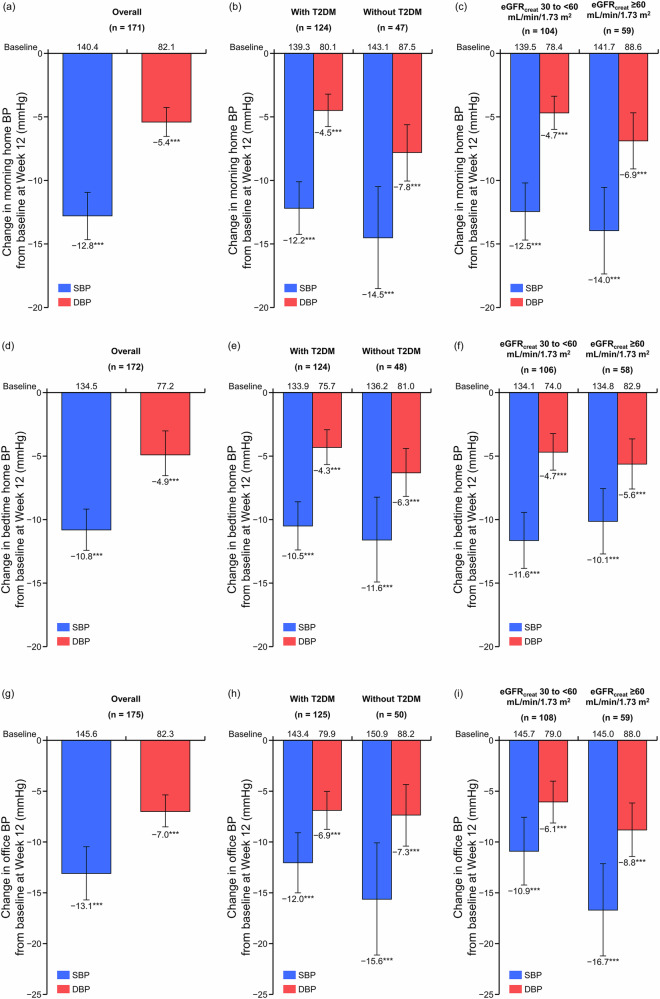
Changes in morning home BP (**a**–**c**), bedtime home BP (**d–f**) and office BP (**g–i**) in the overall population, T2DM subgroups, and eGFR_creat_ subgroups (full analysis set). Mean, error bar (95% CI). ***P < 0.001 vs baseline. *BP* blood pressure, *CI* confidence interval, *DBP* diastolic BP, *eGFR*_creat_ creatinine-based estimated glomerular filtration rate, *SBP* systolic BP, *T2DM* type 2 diabetes mellitus

Figure 1 and Supplementary Table 3 show the changes in morning home BP, bedtime home BP, and office BP from baseline at Week 12 in the overall population and in the T2DM and eGFR_creat_ subgroups. In the overall population, a statistically significant change in morning home SBP/DBP from baseline at Week 12 was observed (mean change: −12.8/ − 5.4 mmHg, *P* < 0.001; Fig. 1a). This BP reduction was consistent in both subgroups with and without T2DM (mean change: −12.2/ − 4.5 and −14.5/ − 7.8 mmHg, respectively, both *P* < 0.001; Fig. 1b). It was also consistent in both subgroups with eGFR_creat_ 30 to <60 and ≥60 mL/min/1.73 m^2^ (mean change: −12.5/ − 4.7 and −14.0/ − 6.9 mmHg, respectively, both *P* < 0.001; Fig. 1c). The bedtime home and office SBP/DBP showed similar tendencies (Fig. 1d–i). The changes in morning home BP, bedtime home BP, and office BP from baseline to Week 12 in the PPS are shown in Supplementary Table 4.

**Fig. 2:**
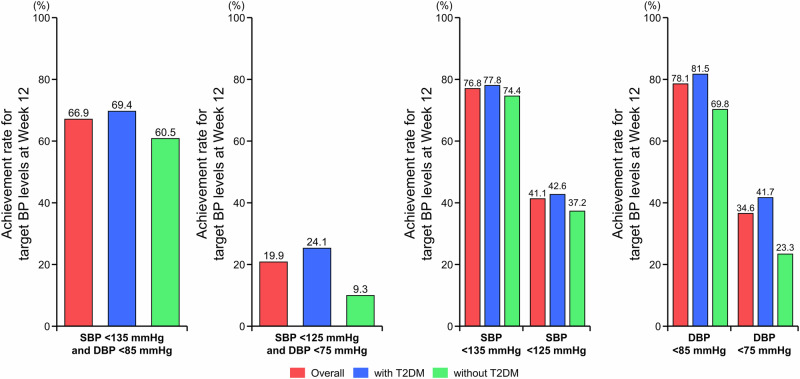
Proportion of patients who reached target morning home BP levels ( < 135/85 mmHg and <125/75 mmHg) at Week 12 in the overall population and in the subgroups with and without T2DM (full analysis set). *BP* blood pressure, *DBP* diastolic BP, *SBP* systolic BP, *T2DM* type 2 diabetes mellitus

The proportions of patients who achieved target BP levels are shown in Fig. 2 and Supplementary Table 5. The proportions of patients who achieved target home SBP/DBP < 135/85 mmHg and office SBP/DBP < 140/90 mmHg in the overall population were as follows: 66.9% for morning home BP, 73.5% for bedtime home BP, and 69.2% for office BP. The proportions of patients who achieved target home SBP/DBP < 125/75 mmHg and office SBP/DBP < 130/80 mmHg in the overall population were as follows: 19.9% for morning home BP, 41.7% for bedtime home BP, and 39.1% for office BP. The percentage of patients who achieved target BP levels was numerically higher in the subgroup with T2DM than in the subgroup without T2DM (no statistical tests were performed). Achievement rates of target BP levels by eGFR_creat_ subgroups were not analyzed.

### Effects on UACR

**Fig. 3:**
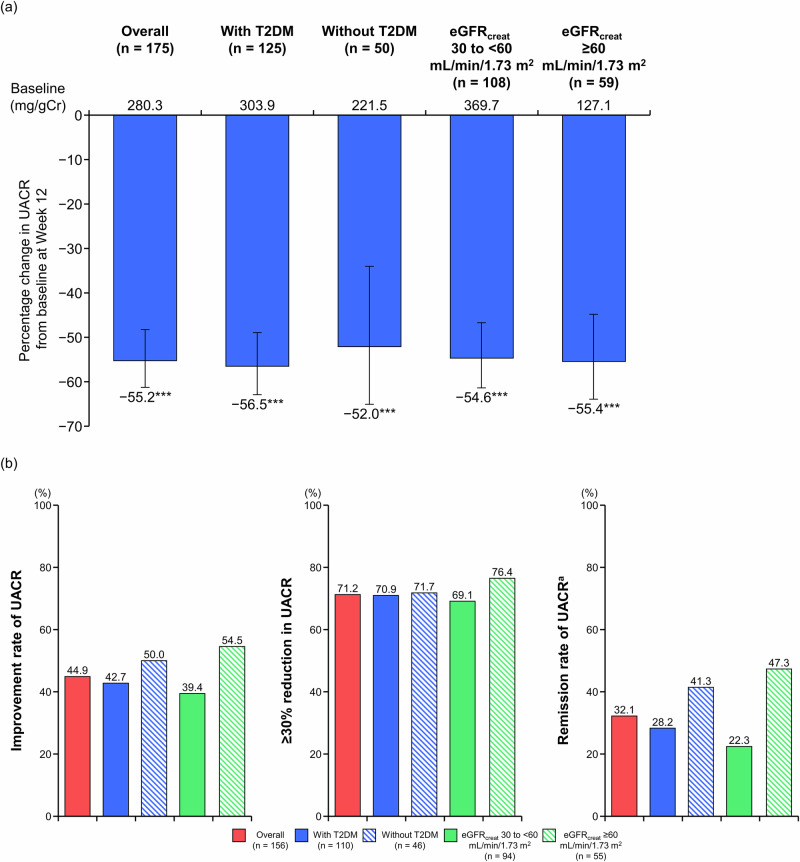
Percentage change in UACR from baseline to Week 12 (**a**) and improvement rates of UACR (**b**) in the overall population, T2DM subgroups, and eGFR_creat_ subgroups (full analysis set). **a** Mean, error bar (95% CI); ***P < 0.001 vs baseline. **b** A2/A3. ^a^Remission was defined as the transition to UACR A1 combined with a ≥ 30% reduction in UACR from baseline. *A1* UACR < 30 mg/gCr, *A2* UACR 30 to <300 mg/gCr, *A3* UACR 300–1000 mg/gCr, *CI* confidence interval, *eGFR*_creat_ creatinine-based estimated glomerular filtration rate, *T2DM* type 2 diabetes mellitus, *UACR* urine albumin-to-creatinine ratio

The UACR showed a statistically significant improvement from baseline at Week 12 in the overall population (mean change: −55.2%, *P* < 0.001); the subgroups with and without T2DM ( − 56.5% and −52.0%), respectively, both *P* < 0.001; and the subgroups with eGFR_creat_ 30 to <60 and ≥60 mL/min/1.73 m^2^ (−54.6% and −55.4%, respectively, both *P* < 0.001) (Fig. 3a and Supplementary Table 6).

The proportion of patients with UACR improvement, ≥30% reduction in UACR, and UACR remission are shown in Fig. 3b and Supplementary Table 7. UACR improved in 44.9% of all patients, 42.7% of patients with T2DM, 50.0% of patients without T2DM, 39.4% of patients with eGFR_creat_ 30 to <60 mL/min/1.73 m^2^, and 54.5% of patients with eGFR_creat_ ≥ 60 mL/min/1.73 m^2^. UACR improvement and UACR remission were similar between T2DM subgroups and between eGFR_creat_ subgroups. The proportion of patients with ≥30% reduction in UACR was 71.2% of all patients, 70.9% of patients with T2DM, 71.7% of patients without T2DM, 69.1% of patients with eGFR_creat_ 30 to <60 mL/min/1.73 m^2^, and 76.4% of patients with eGFR_creat_ ≥ 60 mL/min/1.73 m^2^. In the A2 subcohort, remission of albuminuria was achieved in 41.2% of all patients, 38.2% of patients with T2DM, 47.4% of patients without T2DM, 33.9% of patients with eGFR_creat_ 30 to <60 mL/min/1.73 m^2^, and 51.0% of patients with eGFR_creat_ ≥ 60 mL/min/1.73 m^2^ (Supplementary Table 7). In the A3 subcohort, remission of albuminuria was achieved in 7.1% of all patients, 5.9% of patients with T2DM, 12.5% of patients without T2DM, 2.9% of patients with eGFR_creat_ 30 to <60 mL/min/1.73 m^2^, and 16.7% of patients with eGFR_creat_ ≥ 60 mL/min/1.73 m^2^ (Supplementary Table 7). In the A2 + A3 subcohort, remission of albuminuria was achieved in 32.1% of all patients, 28.2% of patients with T2DM, 41.3% of patients without T2DM, 22.3% of patients with eGFR_creat_ 30 to <60 mL/min/1.73 m^2^, and 47.3% of patients with eGFR_creat_ ≥ 60 mL/min/1.73 m^2^ (Fig. 3b; Supplementary Table 7). Similar tendencies were observed in the PPS (Supplementary Table 8).

NT-proBNP levels significantly decreased from baseline to Week 12 in the overall population (percent change: −14.1%, *P* < 0.001) (Supplementary Table 6). The percent changes in NT-proBNP levels from baseline to Week 12 were −8.7% in the subgroup with T2DM and −25.3% in the subgroup without T2DM, but the change only reached statistical significance in the group without T2DM (*P* < 0.001). NT-proBNP levels by eGFR_creat_ subgroups were not analyzed.

### Safety

The safety results are summarized in Table 2. In the overall population, the incidence of TEAEs was 28.9%; serious TEAEs, 2.2%; ADRs, 8.9%; and serious ADRs, 0%. These results were similar between T2DM subgroups. TEAEs and ADRs by eGFR_creat_ subgroups were not analyzed. In the overall population, the most frequent ADRs were hyperkalemia and blood potassium increased, each in 2.8% of patients. Among the five clinical studies of esaxerenone included in this pooled analysis, no cases of acute kidney injury were reported as TEAEs.

**Table 2:** Safety data (safety analysis set)

	Overall	With T2DM	Without T2DM
	(N = 180)	(n = 127)	(n = 53)
Any TEAE	52 (28.9)	36 (28.4)	16 (30.2)
Serious TEAEs	4 (2.2)	3 (2.4)	1 (1.9)
Cellulitis	1 (0.6)	1 (0.8)	0 (0.0)
Ileus	1 (0.6)	0 (0.0)	1 (1.9)
Upper limb fracture	1 (0.6)	1 (0.8)	0 (0.0)
Glaucoma surgery	1 (0.6)	1 (0.8)	0 (0.0)
Cataract operation	1 (0.6)	1 (0.8)	0 (0.0)
Any ADR	16 (8.9)	9 (7.1)	7 (13.2)
Hyperkalemia	5 (2.8)	1 (0.8)	4 (7.6)
Dizziness	2 (1.1)	2 (1.6)	0 (0.0)
Dizziness postural	1 (0.6)	0 (0.0)	1 (1.9)
Feeling abnormal	1 (0.6)	1 (0.8)	0 (0.0)
Blood potassium increased	5 (2.8)	3 (2.4)	2 (3.8)
Glomerular filtration rate decreased	1 (0.6)	1 (0.8)	0 (0.0)
Liver function test increased	1 (0.6)	1 (0.8)	0 (0.0)
Serious ADRs	0 (0.0)	0 (0.0)	0 (0.0)

Data are n (%)

*ADR* adverse drug reaction, *T2DM* type 2 diabetes mellitus, *TEAE* treatment-emergent adverse event

**Fig. 4:**
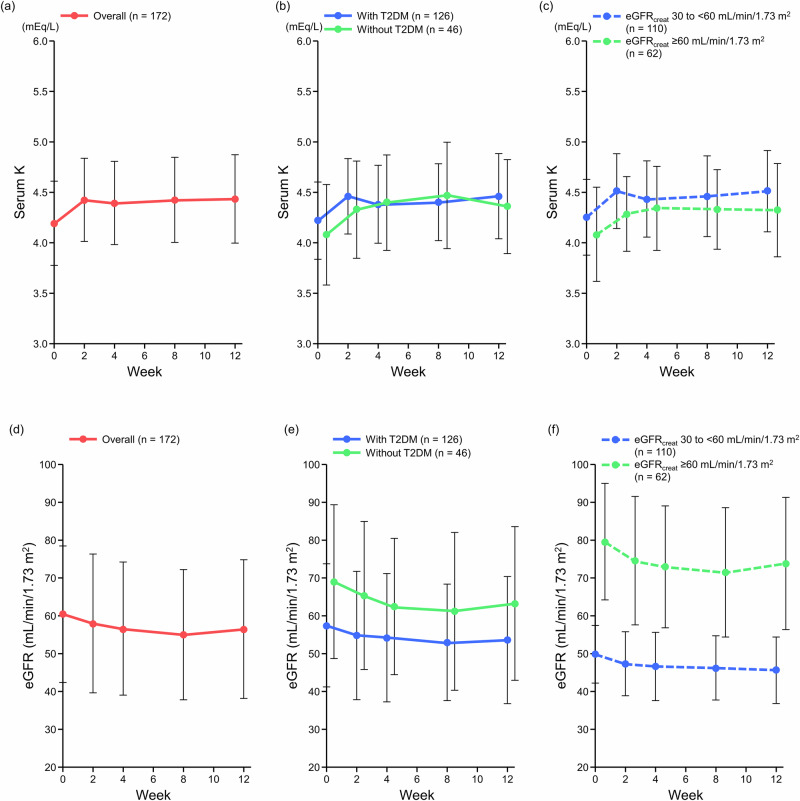
Changes in serum K level (**a–c**) and eGFR_creat_ (**d–f**) in the overall population, T2DM subgroups, and eGFR_creat_ subgroups (safety analysis set). Mean, error bar (standard deviation). *eGFR*_creat_ creatinine-based estimated glomerular filtration rate, *K* potassium, *T2DM* type 2 diabetes mellitus

After starting treatment with esaxerenone, serum K levels increased up to Week 2; thereafter, levels remained stable up to Week 12 (Fig. 4a and Supplementary Table 9). Similar trends were observed in both T2DM subgroups and eGFR_creat_ subgroups (Fig. 4b, c). The incidence of serum K ≥ 5.5 mEq/L was 5.6% (10/180 patients) in the overall population (Supplementary Table 10). The incidence of serum K ≥ 5.5 mEq/L was numerically lower in the subgroup with T2DM (3.1% [4/127 patients]) than in the subgroup without T2DM (11.3% [6/53 patients]); however, no statistical tests were performed. The incidence of serum K ≥ 5.5 mEq/L was slightly higher in patients with eGFR_creat_ 30 to <60 mL/min/1.73 m^2^ (5.5% [6/110 patients]) than in patients with eGFR_creat_ ≥ 60 mL/min/1.73 m^2^ (3.2% [2/62 patients]) (Supplementary Table 10).

After starting esaxerenone treatment, the eGFR_creat_ decreased up to Week 2 and remained stable thereafter up to Week 12 in the overall population, in the T2DM subgroups, and in the eGFR_creat_ subgroups (Fig. 4d–f). The subgroup with T2DM had a lower baseline eGFR_creat_ level than the subgroup without T2DM (Fig. 4e and Supplementary Table 9).

## Discussion

This pooled analysis of five clinical studies showed that esaxerenone significantly lowered morning home BP, bedtime home BP, and office BP in hypertensive patients with albuminuria, regardless of the presence or absence of T2DM and kidney function based on eGFR_creat_ subgroup category (30 to <60 and ≥60 mL/min/1.73 m^2^). Additionally, esaxerenone treatment reduced albuminuria, as evidenced by the significant reduction in UACR in both patient groups, supporting its renoprotective effects. NT-proBNP levels significantly decreased in the overall population and in patients without T2DM, but not in those with T2DM. The overall safety profile of esaxerenone was similar in patients with and without T2DM and in the eGFR_creat_ subgroups. The incidence of serum K ≥ 5.5 mEq/L was numerically higher in patients without T2DM than in those with T2DM, which may be related to the differences in the use of SGLT2is between the two groups.

Previous studies have shown that esaxerenone is effective in reducing BP and albuminuria in hypertensive patients with CKD (albuminuria) [27**–**30]. Our findings are consistent with these results, further confirming the broad antihypertensive efficacy of esaxerenone. Furthermore, our study builds upon these findings by demonstrating that the renoprotective effects of esaxerenone persist regardless of T2DM status.

In this study, esaxerenone was found to significantly lower morning home BP, bedtime home BP, and office BP in hypertensive patients with CKD. The ultimate goal of antihypertensive therapy is to achieve optimal BP control, thus minimizing target organ damage and cardiovascular events [31]. Therefore, achieving consistent 24-hour BP control is important to reduce cardiovascular events [32**–**34]. However, the HI-JAMP study reported that 45%–55% of participants had uncontrolled nocturnal and/or morning hypertension during treatment with three or more antihypertensive drugs [8]. In the present study, esaxerenone lowered BP at all time points (during the morning, office hours, and at bedtime), regardless of the presence or absence of T2DM or kidney function based on eGFR_creat_. This suggests that the BP-lowering effects of esaxerenone are sustained throughout the day across diverse patient profiles. The difference in BP change between subgroups may be influenced by the fact that patients with T2DM and eGFR_creat_ 30 to <60 mL/min/1.73 m^2^ were started on esaxerenone at 1.25 mg, with many still using lower doses at 12 weeks.

Albuminuria was improved and a decrease in UACR was observed. This suggests that esaxerenone has a renoprotective effect, with 55.2% reduction in UACR, independent of T2DM status or kidney function by eGFR_creat_. This reduction of UACR in patients with albuminuria was comparable with the results of previous Phase 3 studies of esaxerenone in T2DM patients with albuminuria, reporting reductions of 32.4% [28], 43.8% [29], and 34.4% at 12 weeks [30]. This reduction in UACR was also comparable with that of previous studies of finerenone, which showed a 31% reduction at 4 months in the FIDELIO-DKD trial [19] and a 32% greater reduction with finerenone versus placebo at 4 months in the FIGARO-DKD trial [18]. Those previous studies showed that finerenone significantly reduced the risk of CKD progression and cardiovascular events compared with placebo [18, 19]. To date, although the long-term effects of esaxerenone on kidney and cardiovascular outcomes have not been examined, its renoprotective effect, based on improvements in UACR and UACR classification, is considered clinically meaningful. Esaxerenone is a selective MRB that works by inhibiting the effects of aldosterone, a hormone that increases BP and promotes kidney damage. The reduction in UACR observed in our study suggests that esaxerenone effectively mitigates aldosterone-induced kidney damage.

NT-proBNP levels decreased significantly in the overall population ( − 14.1%, *P* < 0.001), but the decrease was not statistically significant in patients with T2DM, possibly because of differences in baseline NT-proBNP levels between patients with and without T2DM (123.2 ± 184.0 and 157.5 ± 232.0 pg/mL, respectively); NT-proBNP levels at 12 weeks were similar between the two T2DM subgroups (142.3 ± 300.3 and 134.4 ± 211.4 pg/mL, respectively). It should be noted that in this study, only 47 (26.9%) patients had NT-proBNP levels ≥125 pg/mL at baseline, and the majority were within the normal range. The ESES-LVH study [25], one of the five clinical studies used in this pooled analysis, showed the cardioprotective effects of esaxerenone based on the reduction in NT-proBNP and left ventricular mass index (LVMI) in hypertensive patients with left ventricular hypertrophy. Additionally, recent findings suggest that increased plasma renin activity induced by MRBs, without concurrent RAS inhibition, may be associated with reduced muscle mass in patients with heart failure [35], although muscle wasting and LVMI reduction are distinct phenomena. This may warrant caution when treatment with MRBs is prescribed for patients with heart failure not receiving RAS inhibitors. Thus, further studies are needed to confirm the cardioprotective effects of esaxerenone.

The percentage of patients with serum K ≥ 5.5 mEq/L was higher in patients without T2DM than in those with T2DM (11.3% vs 3.1%). This difference may be due to differences in the frequency of SGLT2i use between patients with and without T2DM, as well as differences in the esaxerenone dose at last administration. Several studies have reported that SGLT2is decrease the risk of hyperkalemia when administered in combination with an MRB including esaxerenone in patients with T2DM [23, 36–39]. Patients with an eGFR_creat_ 30 to <60 mL/min/1.73 m^2^ had a slightly higher incidence of serum K ≥ 5.5 mEq/L than those with eGFR_creat_ ≥ 60 mL/min/1.73 m^2^, despite the lower final dose of esaxerenone. Because reduced kidney function is a known risk factor for hyperkalemia during MRB use, esaxerenone should be administered with greater caution in patients with reduced eGFR_creat_ compared to those with normal kidney function [22].

The results of this study have important implications for the clinical management of hypertensive patients with albuminuria. The ≥10 mmHg reduction in SBP and 50% improvement in UACR are clinically significant findings and may lead to changes in classification (e.g., from macroalbuminuria to microalbuminuria or from microalbuminuria to normoalbuminuria). Although reduction in UACR has not been consistently linked with hard outcomes in some clinical trials, such as ALTITUDE or VA NEPHRON-D [40, 41], reducing proteinuria remains clinically important. While UACR reduction may serve as a potential surrogate marker for renoprotection, definitive evidence linking it to clinical outcomes would require event-driven trials. The present study findings suggest that esaxerenone may offer a valuable treatment option for hypertensive patients with albuminuria, potentially improving outcomes and reducing the risk of cardiovascular and kidney complications. Additionally, the data on eGFR_creat_ and serum K underscore the safety of this protocol. Nevertheless, the cardioprotective effects of esaxerenone need to be verified in future studies.

### Limitations

This study has some limitations that should be considered in the interpretation of its findings. First, data were reported up to 12 weeks only, which may not capture long-term effects. Second, the study population was limited to Japanese patients, and the results may not be generalizable to other populations. Third, this was an analysis of secondary data, which may have introduced bias and influenced the results. Fourth, all findings presented in this study are based on data from single-arm studies, and the lack of comparator groups is a limitation. Fifth, the decision to increase the esaxerenone dose was made by the physician based on the patient’s condition, which may have resulted in a lower rate of achieving antihypertensive control. Sixth, the possibility of type I error should also be considered because corrections for multiplicity were not applied. Seventh, although the study protocol set exclusion criteria to eliminate patients with secondary hypertension, a definitive diagnosis of primary aldosteronism was not performed, so the possibility that patients with primary aldosteronism were included cannot be ruled out. Eighth, only baseline values of plasma aldosterone and renin activity were assessed, and changes following the start of esaxerenone administration were not evaluated. Finally, no statistical tests were performed to compare differences between the patient subgroups.

## Conclusion

The results of this pooled subanalysis demonstrated that esaxerenone significantly lowered morning home, bedtime home, and office BP in hypertensive patients with CKD, regardless of the presence or absence of T2DM. Additionally, esaxerenone treatment improved albuminuria, as evidenced by the significant reduction in UACR in both patient subgroups (by T2DM status and kidney function), supporting its renoprotective effects. NT-proBNP levels significantly decreased in the overall population and in patients without T2DM, but not in those with T2DM, suggesting that the cardioprotective effects of esaxerenone may be limited in patients with T2DM. The overall safety profile of esaxerenone was similar in patients with and without T2DM. The incidence of serum K ≥ 5.5 mEq/L was numerically higher in patients without T2DM than in those with T2DM, which may be related to differences in the use of SGLT2is between the two groups. These findings highlight the efficacy, organ-protective effects, and safety of esaxerenone in hypertensive patients with CKD and warrant further investigation in future studies.

## Supplementary information

Supplementary Materials

Checklist

Disclosure form

## Data Availability

The anonymized data underlying the results presented in this manuscript may be made available to researchers upon submission of a reasonable request to the corresponding author. The decision to disclose the data will be made by the corresponding author and the funder, Daiichi Sankyo Co., Ltd. Data disclosure can be requested for 36 months from article publication.
