# Supplementary material for: Efficacy and safety of esaxerenone in hypertensive patients with chronic kidney disease, with or without type 2 diabetes mellitus: a pooled analysis of five clinical studies
Source: Hypertens Res. 2025 Jun 30;48(9):2413–26. doi: 10.1038/s41440-025-02259-z (PMC12411229; doi:10.1038/s41440-025-02259-z)
Supplement: Supplementary file 1 — Supplementary Materials [file 41440_2025_2259_MOESM1_ESM.docx]

**Supplementary Materials**

**Supplementary Table 1.** Changes in home and office BP and frequency of elevated serum K level in each study

| **Study** | **Target population** | **N patients in FAS** | **Mean morning home BP, mmHg** | | | **UACR** | | **Incidence of serum K**  **≥5.5 mEq/L** |
| --- | --- | --- | --- | --- | --- | --- | --- | --- |
|  |  |  | **Baseline** | **Week 12** | **EOT^1^** | **Mean at baseline** | **<30/ 30 to <300 / ≥300 mg/gCr,**  **n (%)** |  |
| EX-DKD | Hypertensive patients with DKD | 109 | 135.6/75.9 | −12.0/−5.2 | −11.6/−5.2 | 184.0 | 45 (41.3)/ 41 (37.6)/ 23 (21.1) | 3 (2.7) |
| EARLY-NH | Patients with nocturnal hypertension | 93 | 143.8/86.7 | −11.9/−4.2 | −12.2/−4.9 | 110.4 | 65 (69.9)/ 19 (20.4)/ 9 (9.7) | 11 (10.9) |
| ESES-LVH | Hypertensive patients with LVH | 58 | 142.8/85.0 | −10.5/−4.8 | −11.5/−4.7 | NC | NC | 3 (5.0) |
| ENaK | Patients with essential hypertension | 126 | 136.7/88.0 | −12.3/−6.7 | −11.9/−6.4 | 34.6 | 101 (81.5)/ 20 (16.1)/ 3 (2.4) | 9 (7.1) |
| EAGLE-DH | Hypertensive patients with diabetes taking SGLT2i | 93 | 136.4/82.3 | −11.8/−5.1 | −12.1/−5.2 | 145.3 | 58 (62.4)/ 28 (30.1)/ 7 (7.5) | 1 (1.1) |

^1^The dosing period for ESES-LVH and EAGLE-DH was 24 weeks.

Abbreviations: BP, blood pressure; DKD, diabetic kidney disease; EOT, end of treatment; FAS, full analysis set; K, potassium; LVH, left ventricular hypertrophy; NC, not calculated; SGLT2is, sodium–glucose cotransporter-2 inhibitors; UACR urine albumin-to-creatinine ratio.

**Supplementary Table 2.** Patient characteristics (per-protocol set)

|  | **Overall**  **(N = 145)** | **With T2DM**  **(n = 102)** | **Without T2DM**  **(n = 43)** | **eGFR_creat_**  **30 to <60 mL/min/1.73 m^2^**  **(n = 103)** | **eGFR_creat_**  **≥60**  **mL/min/1.73 m^2^**  **(n = 38)** |
| --- | --- | --- | --- | --- | --- |
| Sex, male | 85 (58.6) | 65 (63.7) | 20 (46.5) | 64 (62.1) | 19 (50.0) |
| Age, years | 68.4 ± 11.1 | 69.4 ± 9.5 | 65.9 ± 14.0 | 71.5 ± 8.9 | 60.7 ± 12.7 |
| ≥65 | 102 (70.3) | 78 (76.5) | 24 (55.8) | 85 (82.5) | 16 (42.1) |
| Body mass index, kg/m^2^ | 26.2 ± 4.3  n = 144 | 26.4 ± 3.6  n = 101 | 25.6 ± 5.6  n = 43 | 26.2 ± 3.6  n = 102 | 26.5 ± 5.9  n = 38 |
| ≥25 | 85 (58.6) | 64 (62.7) | 21 (48.8) | 64 (62.1) | 21 (55.3) |
| Current smoker | 32 (22.1) | 21 (20.6) | 11 (25.6) | 18 (17.5) | 13 (34.2) |
| Alcohol use | 62 (42.8) | 41 (40.2) | 21 (48.8) | 39 (37.9) | 21 (55.3) |
| Comorbidities |  |  |  |  |  |
| T2DM | 102 (70.3) | 102 (100.0) | 0 (0.0) | 89 (86.4) | 13 (34.2) |
| Dyslipidemia | 99 (68.3) | 73 (71.6) | 26 (60.5) | 73 (70.9) | 23 (60.5) |
| Hyperuricemia | 52 (35.9) | 33 (32.4) | 19 (44.2) | 39 (37.9) | 10 (26.3) |
| Heart failure | 18 (12.4) | 7 (6.9) | 11 (25.6) | 9 (8.7) | 6 (15.8) |
| Initial dose of esaxerenone |  |  |  |  |  |
| 1.25 mg | 116 (80.0) | 102 (100.0) | 14 (32.6) | 103 (100.0) | 13 (34.2) |
| 2.5 mg | 29 (20.0) | 0 (0.0) | 29 (67.4) | 0 (0.0) | 25 (65.8) |
| Final dose of esaxerenone |  |  |  |  |  |
| 1.25 mg | 50 (34.5) | 45 (44.1) | 5 (11.6) | 45 (43.7) | 5 (13.2) |
| 2.5 mg | 58 (40.0) | 36 (35.3) | 22 (51.2) | 36 (35.0) | 19 (50.0) |
| 5 mg | 37 (25.5) | 21 (20.6) | 16 (37.2) | 22 (21.4) | 14 (36.8) |
| Hypertension disease duration, years | 10.2 ± 9.3  n = 106 | 11.3 ± 9.0  n = 72 | 7.9 ± 9.9  n = 34 | 11.0 ± 10.0  n = 73 | 8.5 ± 7.4  n = 47 |
| Morning home SBP, mmHg | 140.4 ± 12.9  n = 141 | 139.5 ± 12.8  n = 101 | 142.7 ± 13.1  n = 40 | 139.4 ± 12.3  n = 99 | 143.1 ± 14.8  n = 38 |
| Morning home DBP, mmHg | 81.7 ± 12.3  n = 141 | 79.4 ± 11.8  n = 101 | 87.4 ± 11.8  n = 40 | 78.1 ± 11.0  n = 99 | 90.6 ± 11.4  n = 38 |
| Bedtime home SBP, mmHg | 134.3 ± 13.7  n = 142 | 133.9 ± 14.3  n = 101 | 135.5 ± 12.3  n = 41 | 133.8 ± 14.3  n = 101 | 135.4 ± 12.7  n = 37 |
| Bedtime home DBP, mmHg | 76.7 ± 11.7  n = 142 | 75.0 ± 12.1  n = 142 | 80.7 ± 9.6  n = 41 | 73.7 ± 11.5  n = 101 | 84.4 ± 8.7  n = 37 |
| Office SBP, mmHg | 145.7 ± 15.3 | 143.2 ± 13.3 | 151.8 ± 18.0 | 145.6 ± 14.2 | 145.6 ± 18.6 |
| Office DBP, mmHg | 81.5 ± 11.8 | 78.5 ± 9.9 | 88.7 ± 13.0 | 78.7 ± 10.6 | 88.6 ± 12.3 |
| Basal antihypertensive agents |  |  |  |  |  |
| RAS inhibitor | 49 (33.8) | 30 (29.4) | 19 (44.2) | 28 (27.2) | 18 (47.4) |
| CCB | 33 (22.8) | 9 (8.8) | 24 (55.8) | 15 (14.6) | 17 (44.7) |
| Both RAS inhibitor and CCB | 63 (43.4) | 63 (61.8) | 0 (0.0) | 60 (58.3) | 3 (7.9) |
| Diabetes medications |  |  |  |  |  |
| SGLT2i | 54 (37.2) | 54 (52.9) | 0 (0.0) | 48 (46.6) | 6 (15.8) |
| Biguanide | 49 (33.8) | 49 (48.0) | 0 (0.0) | 39 (37.9) | 10 (26.3) |
| Thiazolidinedione | 7 (4.8) | 7 (6.9) | 0 (0.0) | 5 (4.9) | 2 (5.3) |
| Sulfonylurea | 19 (13.1) | 19 (18.6) | 0 (0.0) | 19 (18.4) | 0 (0.0) |
| Glinide | 8 (5.5) | 8 (7.8) | 0 (0.0) | 7 (6.8) | 1 (2.6) |
| DPP-4 inhibitor | 56 (38.6) | 56 (54.9) | 0 (0.0) | 50 (48.5) | 6 (15.8) |
| Alpha glucosidase inhibitor | 17 (11.7) | 17 (16.7) | 0 (0.0) | 14 (13.6) | 3 (7.9) |
| Insulin | 11 (7.6) | 11 (10.8) | 0 (0.0) | 10 (9.7) | 1 (2.6) |
| GLP1 agonist | 5 (3.4) | 5 (4.9) | 0 (0.0) | 4 (3.9) | 1 (2.6) |
| Number of diabetes medications |  |  |  |  |  |
| 1 | 23 (15.9) | 23 (22.5) | 0 (0.0) | 22 (21.4) | 1 (2.6) |
| 2 | 29 (20.0) | 29 (28.4) | 0 (0.0) | 26 (25.2) | 3 (7.9) |
| 3 | 42 (29.0) | 42 (41.2) | 0 (0.0) | 35 (34.0) | 7 (18.4) |
| None | 51 (35.2) | 8 (7.8) | 43 (100.0) | 20 (19.4) | 27 (71.1) |
| NT-proBNP, pg/mL | 124.8 ± 183.9  80.4 (37.0, 126.0)  n = 141 | 126.1 ± 192.4  84.6 (37.0, 130.0)  n = 98 | 122.0 ± 165.1  68.7 (36.8, 125.0)  n = 43 | 145.3 ± 208.3  91.0 (46.5, 143.0)  n = 99 | 75.9 ± 94.2  39.9 (27.3, 80.4)  n = 38 |
| <125 | 104 (71.7) | 72 (70.6) | 32 (74.4) | 68 (66.0) | 33 (86.8) |
| ≥125 | 37 (25.5) | 26 (25.5) | 11 (25.6) | 31 (30.1) | 5 (13.2) |
| UACR, mg/gCr | 312.9 ± 580.3  136.5 (54.3, 358.0) | 345.6 ± 630.4  170.2 (66.7, 451.8) | 235.4 ± 435.9  72.4(38.5, 207.2) | 381.0 ± 664.6  180.1 (72.6, 488.6) | 144.0 ± 210.7  48.0 (35.7, 139.0) |
| ≥30 | 145 (100.0) | 102 (100.0) | 43 (100.0) | 103 (100.0) | 38 (100.0) |
| Serum potassium, mEq/L | 4.2 ± 0.4  n = 141 | 4.3 ± 0.3  n = 102 | 4.0 ± 0.5  n = 39 | 4.2 ± 0.4  n = 103 | 4.1 ± 0.4  n = 38 |
| <4.5 | 104 (71.7) | 75 (73.5) | 29 (67.4) | 73 (70.9) | 31 (81.6) |
| ≥4.5 | 37 (25.5) | 27 (26.5) | 10 (23.3) | 30 (29.1) | 7 (18.4) |
| eGFR_creat_, mL/min/1.73 m^2^ | 57.9 ± 17.2  n = 141 | 53.2 ± 13.1  n = 102 | 70.3 ± 20.4  n = 39 | 49.6 ± 7.6  n = 103 | 80.3 ± 15.9  n = 38 |
| 30 to <60 | 103 (71.0) | 89 (87.3) | 14 (32.6) | 103 (100.0) | 0 (0.0) |
| ≥60 | 38 (26.2) | 13 (12.7) | 25 (58.1) | 0 (0.0) | 38 (100.0) |
| Plasma aldosterone, pg/mL | 57.8 ± 40.4  n = 130 | 65.0 ± 42.1  n = 96 | 37.6 ± 26.4  n = 34 | 59.4 ± 34.2  n = 98 | 56.4 ± 58.5  n = 28 |
| <120 | 123 (84.8) | 90 (88.2) | 33 (76.7) | 94 (91.3) | 25 (65.8) |
| ≥120 | 7 (4.8) | 6 (5.9) | 1 (2.3) | 4 (3.9) | 3 (7.9) |
| Plasma renin activity, ng/mL/h | 3.0 ± 4.0  n = 136 | 3.5 ± 4.5  n = 97 | 1.7 ± 2.0  n = 39 | 3.0 ± 3.2  n = 99 | 3.2 ± 6.0  n = 33 |
| <1.0 | 44 (30.3) | 25 (24.5) | 19 (44.2) | 28 (27.2) | 15 (39.5) |
| ≥1.0 | 92 (63.4) | 72 (70.6) | 20 (46.5) | 71 (68.9) | 18 (47.4) |

Data are n (%), mean ± standard deviation, or median (Q1, Q3).

Abbreviations: CCB, calcium channel blocker; DBP, diastolic blood pressure; DPP-4, dipeptidyl peptidase-4; eGFR_creat_, creatinine-based estimated glomerular filtration rate; GLP1, glucagon-like peptide-1; NT-proBNP, N-terminal prohormone of brain natriuretic peptide; Q, quartile; RAS, renin–angiotensin system; SBP, systolic blood pressure; SGLT2i, sodium–glucose cotransporter 2 inhibitor; T2DM, type 2 diabetes mellitus; UACR, urine albumin-to-creatinine ratio.

**Supplementary Table 3.** Changes in morning home BP, bedtime home BP, and office BP (full analysis set)

|  | **Overall** | | | **With T2DM** | | | **Without T2DM** | | | **eGFR_creat_ 30 to <60 mL/min/1.73 m^2^** | | | **eGFR_creat_ ≥60 mL/min/1.73 m^2^** | | |
| --- | --- | --- | --- | --- | --- | --- | --- | --- | --- | --- | --- | --- | --- | --- | --- |
| **Morning home BP** | **n** | **SBP, mmHg** | **DBP, mmHg** | **n** | **SBP, mmHg** | **DBP, mmHg** | **n** | **SBP, mmHg** | **DBP, mmHg** | **n** | **SBP, mmHg** | **DBP, mmHg** | **n** | **SBP, mmHg** | **DBP, mmHg** |
| Baseline | 171 | 140.4 ± 12.7 | 82.1 ± 11.7 | 124 | 139.3 ± 12.7 | 80.1 ± 11.2 | 47 | 143.1 ± 12.4 | 87.5 ± 11.3 | 104 | 139.5 ± 12.2 | 78.4 ± 11.1 | 59 | 141.7 ± 14.1 | 88.6 ± 10.4 |
| Week 2 | 131 | 136.3 ± 13.6 | 81.0 ± 12.0 | 83 | 135.6 ± 12.7 | 79.2 ± 12.1 | 48 | 137.5 ± 15.1 | 84.3 ± 11.3 |  |  |  |  |  |  |
| Change from baseline | 127 | −5.4 ± 8.8***  [−7.0, −3.9] | −1.7 ± 5.1***  [−2.6, −0.8] | 82 | −4.5 ± 9.3***  [−6.6, −2.5] | −0.7 ± 5.5  [−1.9, 0.5] | 45 | −7.1 ± 7.7***  [−9.4, −4.8] | −3.6 ± 3.5***  [−4.6, −2.6] |  |  |  |  |  |  |
| Week 4 | 161 | 133.1 ± 12.6 | 79.3 ± 10.4 | 115 | 132.7 ± 12.3 | 78.4 ± 10.4 | 46 | 134.2 ± 13.6 | 81.7 ± 10.1 |  |  |  |  |  |  |
| Change from baseline | 159 | −7.8 ± 8.6***  [−9.1, −6.4] | −2.9 ± 5.1***  [−3.7, −2.1] | 115 | −6.9 ± 8.2***  [−8.4, −5.4] | −2.0 ± 4.7***  [−2.9, −1.2] | 44 | −10.0 ± 9.2***  [−12.8, −7.2] | −5.3 ± 5.4***  [−6.9, −3.7] |  |  |  |  |  |  |
| Week 8 | 154 | 130.4 ± 12.9 | 78.0 ± 10.8 | 109 | 130.3 ± 12.7 | 77.2 ± 10.4 | 45 | 130.8 ± 13.4 | 80.0 ± 11.4 |  |  |  |  |  |  |
| Change from baseline | 152 | −10.9 ± 10.2***  [−12.5, −9.3] | −4.5 ± 6.0***  [−5.5, −3.6] | 109 | −9.7 ± 10.3***  [−11.7, −7.8] | −3.4 ± 5.7***  [−4.5, −2.3] | 43 | −13.9 ± 9.4***  [−16.8, −11.0] | −7.4 ± 5.9***  [−9.2, −5.6] |  |  |  |  |  |  |
| Week 12 | 151 | 128.6 ± 13.2 | 77.4 ± 10.9 | 108 | 128.0 ± 12.2 | 76.4 ± 10.1 | 43 | 130.1 ± 15.3 | 80.1 ± 12.3 | 91 | 128.4 ± 13.4 | 74.3 ± 10.6 | 53 | 128.2 ± 12.4 | 82.5 ± 9.5 |
| Change from baseline | 149 | −12.8 ± 11.4***  [−14.7, −11.0] | −5.4 ± 7.0***  [−6.5, −4.2] | 108 | −12.2 ± 10.9***  [−14.2, −10.1] | −4.5 ± 6.7***  [−5.7, −3.2] | 41 | −14.5 ± 12.8***  [−18.5, −10.5] | −7.8 ± 7.1***  [−10.0, −5.5] | 89 | −12.5 ± 10.8***  [−14.7, −10.2] | −4.7 ± 6.2***  [−6.0, −3.4] | 53 | −14.0 ± 12.4***  [−17.4, −10.6] | −6.9 ± 8.0***  [−9.1, −4.7] |
| **Bedtime home BP** | **n** | **SBP, mmHg** | **DBP, mmHg** | **n** | **SBP, mmHg** | **DBP, mmHg** | **n** | **SBP, mmHg** | **DBP, mmHg** | **n** | **SBP, mmHg** | **DBP, mmHg** | **n** | **SBP, mmHg** | **DBP, mmHg** |
| Baseline | 172 | 134.5 ± 14.0 | 77.2 ± 11.7 | 124 | 133.9 ± 14.5 | 75.7 ± 12.0 | 48 | 136.2 ± 12.5 | 81.0 ± 9.9 | 106 | 134.1 ± 14.2 | 74.0 ± 11.8 | 58 | 134.8 ± 14.0 | 82.9 ± 9.4 |
| Week 2 | 131 | 130.0 ± 14.6 | 75.8 ± 11.7 | 83 | 129.8 ± 14.5 | 74.3 ± 11.6 | 48 | 130.3 ± 15.0 | 78.4 ± 11.7 |  |  |  |  |  |  |
| Change from baseline | 128 | −5.5 ± 8.1***  [−6.9, −4.1] | −2.0 ± 4.9***  [−2.9, −1.2] | 82 | −4.8 ± 7.7***  [−6.6, −3.2] | −1.4 ± 4.7**  [−2.5, −0.4] | 46 | −6.7 ± 8.7***  [−9.3, −4.1] | −3.1 ± 5.2***  [−4.7, −1.6] |  |  |  |  |  |  |
| Week 4 | 161 | 127.9 ± 14.1 | 75.0 ± 10.9 | 115 | 127.8 ± 14.0 | 74.2 ± 11.4 | 46 | 127.9 ± 14.3 | 76.9 ± 9.5 |  |  |  |  |  |  |
| Change from baseline | 159 | −7.1 ± 8.2***  [−8.4, −5.9] | −2.7 ± 5.7***  [−3.6, −1.8] | 115 | −6.4 ± 8.1***  [−7.9, −4.9] | −1.9 ± 5.5***  [−2.9, −0.9] | 44 | −9.1 ± 8.1***  [−11.6, −6.7] | −4.7 ± 5.9***  [−6.5, −2.9] |  |  |  |  |  |  |
| Week 8 | 154 | 125.9 ± 13.9 | 73.7 ± 11.4 | 109 | 125.5 ± 14.2 | 72.6 ± 11.3 | 45 | 126.8 ± 13.4 | 76. 2 ± 11.3 |  |  |  |  |  |  |
| Change from baseline | 152 | −9.4 ± 10.2***  [−11.0, −7.7] | −3.9 ± 6.5***  [−5.0, −2.9] | 109 | −8.8 ± 10.2***  [−10.8, −6.9] | −3.5 ± 6.4***  [−4.8, −2.3] | 43 | −10.7 ± 10.1***  [−13.8, −7.6] | −5.0 ± 6.7***  [−7.0, −2.9] |  |  |  |  |  |  |
| Week 12 | 151 | 124.4 ± 14.4 | 73.1 ±  11.5 | 108 | 124.0 ± 13.4 | 72.3 ± 11.1 | 43 | 125.1 ± 16.6 | 75.0 ± 12.4 | 91 | 123.6 ± 14.2 | 70.2 ± 11.7 | 53 | 124.5 ± 14.5 | 77.7 ± 9.8 |
| Change from baseline | 150 | −10.8 ± 10.2***  [−12.5, −9.2] | −4.9 ± 6.9***  [−6.0, −3.7] | 108 | −10.5 ± 10.0***  [−12.4, −8.6] | −4.3 ± 7.2***  [−5.7, −2.9] | 42 | −11.6 ± 10.7***  [−14.9, −8.2] | −6.3 ± 6.0***  [−8.2, −4.4] | 90 | −11.6 ± 10.5***  [−13.8, −9.4] | −4.7 ± 6.9***  [−6.1, −3.2] | 53 | −10.1 ± 9.3***  [−12.7, −7.5] | −5.6 ± 7.1***  [−7.6, −3.7] |
| **Office BP** | **n** | **SBP, mmHg** | **DBP, mmHg** | **n** | **SBP, mmHg** | **DBP, mmHg** | **n** | **SBP, mmHg** | **DBP, mmHg** | **n** | **SBP, mmHg** | **DBP, mmHg** | **n** | **SBP, mmHg** | **DBP, mmHg** |
| Baseline | 175 | 145.6 ± 15.3 | 82.3 ± 11.6 | 125 | 143.4 ± 13.7 | 79.9 ± 10.4 | 50 | 150.9 ± 17.7 | 88.2 ± 12.6 | 108 | 145.7 ± 13.9 | 79.0 ± 10.8 | 59 | 145.0 ± 17.7 | 88.0 ± 11.5 |
| Week 2 | 67 | 137.0 ± 15.1 | 75.0 ± 10.5 | 62 | 137.3 ± 15.2 | 73.5 ± 9.1 | 5 | 134.2 ± 15.6 | 93.6 ± 9.3 |  |  |  |  |  |  |
| Change from baseline | 67 | −7.4 ± 13.9***  [−10.8, −4.0] | −3.1 ± 9.3**  [−5.3, −0.8] | 62 | −7.6 ± 13.2***  [−10.9, −4.2] | −3.5 ± 8.9**  [−5.7, −1.2] | 5 | −5.6 ± 22.1  [−33.1, 21.9] | 2.2 ± 12.8  [−13.7, 18.1] |  |  |  |  |  |  |
| Week 4 | 163 | 135.8 ± 14.5 | 78.6 ± 11.3 | 115 | 135.1 ± 14.2 | 76.7 ± 9.9 | 48 | 137.7 ± 15.1 | 83.3 ± 13.0 |  |  |  |  |  |  |
| Change from baseline | 163 | −9.2 ± 15.0***  [−11.6, −6.9] | −3.5 ± 9.5***  [−4.9, −2.0] | 115 | −7.8 ± 13.8***  [−10.3, −5.2] | −3.1 ± 9.7***  [−4.9, −1.3] | 48 | −12.8 ± 17.3***  [−17.8, −7.7] | −4.4 ± 9.0**  [−7.0, −1.8] |  |  |  |  |  |  |
| Week 8 | 147 | 134.6 ± 16.0 | 76.2 ± 12.4 | 110 | 132.7 ± 14.4 | 74.0 ± 11.4 | 37 | 140.3 ± 19.0 | 82.6 ± 13.1 |  |  |  |  |  |  |
| Change from baseline | 147 | −10.4 ± 13.5***  [−12.6, −8.2] | −5.6 ± 9.5***  [−7.1, −4.0] | 110 | −9.9 ± 13.1***  [−12.3, −7.4] | −5.5 ± 9.9***  [−7.4, −3.6] | 37 | −11.8 ± 14.6***  [−16.7, −7.0] | −5.7 ± 8.3***  [−8.5, −3.0] |  |  |  |  |  |  |
| Week 12 | 156 | 131.9 ± 16.4 | 75.1 ± 12.0 | 110 | 130.6 ± 16.0 | 72.9 ± 11.0 | 46 | 135.0 ± 17.1 | 80.2 ± 13.0 | 94 | 134.2 ± 16.9 | 72.7 ± 12.1 | 55 | 128.0 ± 13.9 | 78.8 ± 11.3 |
| Change from baseline | 156 | −13.1 ± 16.6***  [−15.7, −10.5] | −7.0 ± 9.9***  [−8.6, −5.4] | 110 | −12.0 ± 15.6***  [−15.0, −9.1] | −6.9 ± 9.9***  [−8.7, −5.0] | 46 | −15.6 ± 18.6***  [−21.1, −10.1] | −7.3 ± 10.2***  [−10.4, −4.3] | 94 | −10.9 ± 16.3***  [−14.2, −7.6] | −6.1 ± 10.0***  [−8.1, −4.0] | 55 | −16.7 ± 16.7***  [−21.2, −12.2] | −8.8 ± 9.7***  [−11.4, −6.2] |

Data are mean ± standard deviation [95% CI].

***P* <0.01 versus baseline, paired *t*-test.

****P* <0.001 versus baseline, paired *t*-test.

Abbreviations: BP, blood pressure; CI, confidence interval; DBP, diastolic BP; eGFR_creat_, creatinine-based estimated glomerular filtration rate; SBP, systolic BP; T2DM, type 2 diabetes mellitus.

**Supplementary Table 4.** Changes in morning home BP, bedtime home BP, and office BP (per-protocol set)

|  | **Overall** | | | **With T2DM** | | | **Without T2DM** | | | **eGFR_creat_ 30 to <60 mL/min/1.73 m^2^** | | | **eGFR_creat_ ≥60 mL/min/1.73 m^2^** | | |
| --- | --- | --- | --- | --- | --- | --- | --- | --- | --- | --- | --- | --- | --- | --- | --- |
| **Morning home BP** | **n** | **SBP, mmHg** | **DBP, mmHg** | **n** | **SBP, mmHg** | **DBP, mmHg** | **n** | **SBP, mmHg** | **DBP, mmHg** | **n** | **SBP, mmHg** | **DBP, mmHg** | **n** | **SBP, mmHg** | **DBP, mmHg** |
| Baseline | 141 | 140.4 ± 12.9 | 81.7 ± 12.3 | 101 | 139.5 ± 12.8 | 79.4 ± 11.8 | 40 | 142.7 ± 13.1 | 87.4 ± 11.8 | 99 | 139.4 ± 12.3 | 78.1 ± 11.0 | 38 | 143.1 ± 14.8 | 90.6 ± 11.4 |
| Week 2 | 115 | 136.0 ± 14.0 | 80.7 ± 12.3 | 73 | 135.3 ± 12.8 | 78.5 ± 12.2 | 42 | 137.3 ± 16.0 | 84.5 ± 11.8 |  |  |  |  |  |  |
| Change from baseline | 111 | −5.4 ± 8.5  [−7.0, −3.8]*** | −1.7 ± 4.8  [−2.6, −0.8]*** | 72 | −4.4 ± 8.8  [−6.5, −2.4]*** | −0.7 ± 5.1  [−1.9, 0.4] | 39 | −7.1 ± 7.7  [−9.6, −4.6]*** | −3.5 ± 3.6  [−4.7, −2.3]*** |  |  |  |  |  |  |
| Week 4 | 132 | 133.8 ± 13.1 | 78.9 ± 11.0 | 93 | 133.5 ± 12.5 | 77.7 ± 11.0 | 39 | 134.6 ± 14.6 | 81.7 ± 10.8 |  |  |  |  |  |  |
| Change from baseline | 130 | −6.9 ± 8.4  [−8.4, −5.5]*** | −2.7 ± 5.1  [−3.6, −1.8]*** | 93 | −6.0 ± 7.9  [−7.6, −4.4]*** | −1.7 ± 4.6  [−2.7, −0.8]*** | 37 | −9.3 ± 9.2  [−12.4, −6.2]*** | −5.1 ± 5.6  [−7.0, −3.2]*** |  |  |  |  |  |  |
| Week 8 | 127 | 130.9 ± 13.3 | 77.7 ± 11.4 | 89 | 130.8 ± 12.9 | 76.8 ± 11.1 | 38 | 131.2 ± 14.4 | 79.9 ± 12.2 |  |  |  |  |  |  |
| Change from baseline | 125 | −10.3 ± 9.6  [−12.0, −8.6]*** | −4.3 ± 5.9  [−5.3, −3.2]*** | 89 | −9.1 ± 9.5  [−11.1, −7.1]*** | −3.0 ± 5.3  [−4.1, −1.9]*** | 36 | −13.3 ± 9.2  [−16.4, −10.2]*** | −7.4 ± 6.1  [−9.5, −5.4]*** |  |  |  |  |  |  |
| Week 12 | 123 | 128.5 ± 13.7 | 76.6 ± 11.23 | 87 | 128.0 ± 12.6 | 75.3 ± 10.3 | 36 | 129.8 ± 16.2 | 79.8 ± 13.0 | 88 | 128.3 ± 13.4 | 74.0 ± 10.6 | 32 | 128.5 ± 13.5 | 83.3 ± 10.3 |
| Change from baseline | 121 | −12.8 ± 11.1  [−14.8, −10.8]*** | −5.6 ± 6.6  [−6.8, −4.4]*** | 87 | −12.0 ± 10.4  [−14.2, −9.8]*** | −4.6 ± 6.1  [−5.9, −3.3]*** | 34 | −14.7 ± 12.6  [−19.1, −10.3]*** | −8.2 ± 7.2  [−10.7, −5.7]*** | 86 | −12.4 ± 10.3  [−14.6, −10.2]*** | −4.6 ± 6.1  [−5.9, −3.3]*** | 32 | −14.6 ± 12.1  [−19.0, −10.3]*** | −8.8 ± 6.9  [−11.2, −6.3]*** |
| **Bedtime home BP** | **n** | **SBP, mmHg** | **DBP, mmHg** | **n** | **SBP, mmHg** | **DBP, mmHg** | **n** | **SBP, mmHg** | **DBP, mmHg** | **n** | **SBP, mmHg** | **DBP, mmHg** | **n** | **SBP, mmHg** | **DBP, mmHg** |
| Baseline | 142 | 134.3 ± 13.7 | 76.7 ± 11.7 | 101 | 133.9 ± 14.3 | 75.0 ± 12.1 | 41 | 135.5 ± 12.3 | 80.7 ± 9.6 | 101 | 133.8 ± 14.3 | 73.7 ± 11.5 | 37 | 135.4± 12.7 | 84.4 ± 8.7 |
| Week 2 | 115 | 129.5 ± 14.7 | 75.2 ± 11.9 | 73 | 128.8 ± 14.2 | 73.2 ± 11.4 | 42 | 130.8 ± 15.6 | 78.7 ± 12.0 |  |  |  |  |  |  |
| Change from baseline | 112 | −5.5 ± 7.7  [−6.9, −4.0]*** | −2.1 ± 4.8  [−3.0, −1.2]*** | 72 | −5.3 ± 7.1  [−7.0, −3.7]*** | −1.8 ± 4.4  [−2.8, −0.7]*** | 40 | −5.8 ± 8.7  [−8.5, −3.0]*** | −2.8 ± 5.4  [−4.5, −1.0]** |  |  |  |  |  |  |
| Week 4 | 132 | 128.0 ±14.4 | 74.5 ± 11.1 | 93 | 128.0 ± 14.2 | 73.4 ± 11.6 | 39 | 128.1 ± 14.8 | 77.1 ± 9.7 |  |  |  |  |  |  |
| Change from baseline | 130 | −6.7 ± 7.9  [−8.1, −5.4]*** | −2.6 ± 5.7  [−3.6, −1.6]*** | 93 | −6.1 ± 7.8  [−7.7, −4.5]*** | −1.9 ± 5.5  [−3.0, −0.7]** | 37 | −8.4 ± 7.9  [−11.0, −5.7]*** | −4.3 ± 5.9  [−6.3, −2.3]*** |  |  |  |  |  |  |
| Week 8 | 127 | 125.8 ± 14.0 | 73.2 ± 11.7 | 89 | 125.6 ± 14.1 | 72.0 ± 11.5 | 38 | 126.5 ± 13.9 | 76.0 ± 11.7 |  |  |  |  |  |  |
| Change from baseline | 125 | −9.2 ± 10.1  [−11.0, −7.5]*** | −3.9 ± 6.5  [−5.1, −2.8]*** | 89 | −8.8 ± 10.0  [−10.9, −6.7]*** | −3.6 ± 6.3  [−4.9, −2.3]*** | 36 | −10.4 ± 10.5  [−13.9, −6.8]*** | −4.8 ± 7.1  [−7.2, −2.4]*** |  |  |  |  |  |  |
| Week 12 | 123 | 123.8 ± 14.6 | 71.9 ± 11.7 | 87 | 123.6 ± 13.3 | 70.9 ± 11.0 | 36 | 124.3 ± 17.4 | 74.3 ± 13.0 | 88 | 123.3 ± 14.2 | 69.7 ± 11.5 | 32 | 124.0 ± 14.6 | 77.3 ± 10.1 |
| Change from baseline | 122 | −11.1 ± 10.3  [−12.9, −9.2]*** | −5.3 ± 6.8  [−6.5, −4.1]*** | 87 | −10.8 ± 9.9  [−12.9, −8.7]*** | −4.7 ± 7.0  [−6.2, −3.2]*** | 35 | −11.7 ± 11.2 [−15.6, −7.9]*** | −6.8 ± 6.1  [−8.9, −4.7]*** | 87 | −11.4 ± 10.6  [−13.7, −9.2]*** | −4.6 ± 7.0  [−6.0, −3.1]*** | 32 | −10.8 ± 8.8  [−14.0, −7.6]*** | −7.6 ± 5.9  [−9.7, −5.5]*** |
| **Office BP** | **n** | **SBP, mmHg** | **DBP, mmHg** | **n** | **SBP, mmHg** | **DBP, mmHg** | **n** | **SBP, mmHg** | **DBP, mmHg** | **n** | **SBP, mmHg** | **DBP, mmHg** | **n** | **SBP, mmHg** | **DBP, mmHg** |
| Baseline | 145 | 145.7 ± 15.3 | 81.5 ± 11.8 | 102 | 143.2 ± 13.3 | 78.5 ± 9.9 | 43 | 151.8 ± 18.0 | 88.7 ± 13.0 | 103 | 145.6 ± 14.2 | 78.7 ± 10.6 | 38 | 145.6 ± 18.6 | 88.6 ± 12.3 |
| Week 2 | 63 | 137.5 ± 15.2 | 74.5 ± 10.4 | 60 | 137.2 ± 15.4 | 73.5 ± 9.2 | 3 | 142.7 ± 13.2 | 95.7 ± 12.5 |  |  |  |  |  |  |
| Change from baseline | 63 | −6.7 ± 13.8  [−10.2, −3.2]*** | −2.9 ± 9.3  [−5.2, −0.5]* | 60 | −7.5 ± 13.4  [−10.9, −4.0]*** | −3.3 ± 9.0  [−5.6, −1.0]** | 3 | 8.0 ± 16.4  [−32.7, 48.7] | 5.3 ± 12.7  [−26.1, 36.8] |  |  |  |  |  |  |
| Week 4 | 134 | 136.9 ± 14.8 | 78.3 ± 11.5 | 93 | 136.4 ± 14.8 | 76.2 ± 9.6 | 41 | 138.1 ± 15.1 | 83.3 ± 13.9 |  |  |  |  |  |  |
| Change from baseline | 134 | −8.4 ± 15.2  [−11.0, −5.8]*** | −2.9 ± 9.4  [−4.5, −1.2]*** | 93 | −6.3 ± 13.3  [−9.1, −3.6]*** | −2.0 ± 9.4  [−3.9, −0.0]* | 41 | −13.1 ± 18.3  [−18.9, −7.4]*** | −4.9 ± 9.2  [−7.7, −2.0]** |  |  |  |  |  |  |
| Week 8 | 124 | 135.7 ± 16.1 | 75.7 ± 12.5 | 90 | 133.8 ± 14.3 | 72.9 ± 11.0 | 34 | 140.5 ± 19.5 | 83.3 ± 13.4 |  |  |  |  |  |  |
| Change from baseline | 124 | −9.6 ± 13.2  [−11.9, −7.3]*** | −5.3 ± 8.8  [−6.9, −3.8]*** | 90 | −8.6 ± 12.3  [−11.2, −6.0]*** | −5.2 ± 9.0  [−7.1, −3.3]*** | 34 | −12.3 ± 15.0  [−17.5, −7.0]*** | −5.7 ± 8.3  [−8.6, −2.8]*** |  |  |  |  |  |  |
| Week 12 | 128 | 132.6 ± 16.2 | 74.3 ± 12.3 | 89 | 131.9 ± 15.9 | 72.0 ± 11.2 | 39 | 134.1 ± 16.9 | 79.6 ± 13.3 | 91 | 134.2 ± 17.1 | 72.1 ± 11.7 | 34 | 129.4 ± 12.8 | 79.9 ± 12.4 |
| Change from baseline | 128 | −12.6 ± 16.4  [−15.5, −9.8]*** | −6.8 ± 10.0  [−8.6, −5.1]*** | 89 | −10.6 ± 14.6  [−13.7, −7.5]*** | −6.2 ± 9.8  [−8.2, −4.1]*** | 39 | −17.4 ± 19.3  [−23.6, −11.1]*** | −8.3 ± 10.3  [−11.6, −4.9]*** | 91 | −10.9 ± 16.5  [−14.3, −7.5]*** | −6.2 ± 10.1  [−8.3, −4.1]*** | 34 | −16.4 ± 15.9  [−22.0, −10.9]*** | −8.3 ± 9.8  [−11.7, −4.9]*** |

Data are mean ± standard deviation [95% CI].

**P* <0.05 versus baseline, paired *t*-test.

***P* <0.01 versus baseline, paired *t*-test.

****P* <0.001 versus baseline, paired *t*-test.

Abbreviations: BP, blood pressure; CI, confidence interval; eGFR_creat_, creatinine-based estimated glomerular filtration rate; Q, quartile; T2DM, type 2 diabetes mellitus.

**Supplementary Table 5.** Achievement rate (%) of target BP levels at Week 12 (FAS and PPS)

|  | **FAS** | | | **PPS** | | |
| --- | --- | --- | --- | --- | --- | --- |
|  | **Overall**  **(N = 151）** | **With T2DM**  **(n = 108）** | **Without T2DM**  **(n = 43）** | **Overall**  **(N = 123）** | **With T2DM**  **(n = 87）** | **Without T2DM**  **(n = 36）** |
| **Target: home SBP/DBP <135/85 mmHg; office SBP/DBP <140/90 mmHg** | | | | | | |
| Morning home SBP/DBP | 66.9  [58.8, 74.3] | 69.4  [59.8, 77.9] | 60.5  [44.4, 75.0] | 66.7  [57.6, 74.9] | 67.8  [56.9, 77.4] | 63.9  [46.2, 79.2] |
| Bedtime home SBP/DBP | 73.5  [65.7, 80.4] | 75.9  [66.7, 83.6] | 67.4  [51.5, 80.9] | 74.8  [66.2, 82.2] | 75.9  [65.5, 84.4] | 72.2  [54.8, 85.8] |
| Office SBP/DBP | 69.2  [61.4, 76.4] | 71.8  [62.4, 80.0] | 63.0  [47.5, 76.8] | 67.2  [58.3, 75.2] | 68.5  [57.8, 78.0] | 64.1  [47.2, 78.8] |
| **Target: home SBP <135 mmHg; office SBP <140 mmHg** | | | | | | |
| Morning home SBP | 76.8  [69.3, 83.3] | 77.8  [68.8, 85.2] | 74.4  [58.8, 86.5] | 75.6  [67.0, 82.9] | 75.9  [65.5, 84.4] | 75.0  [57.8, 87.9] |
| Bedtime home SBP | 78.8  [71.4, 85.0] | 81.5  [72.9, 88.3] | 72.1  [56.3,84.7] | 79.7  [71.5, 86.4] | 81.6  [71.9, 89.1] | 75.0  [57.8, 87.9] |
| Office SBP | 71.2  [63.4, 78.1] | 72.7  [63.4, 80.8] | 67.4  [52.0, 80.5] | 69.5  [60.8, 77.4] | 69.7  [59.0, 79.0] | 69.2  [52.4, 83.0] |
| **Target: home DBP <85 mmHg; office DBP <90 mmHg** | | | | | | |
| Morning home DBP | 78.1  [70.7, 84.5] | 81.5  [72.9, 88.3] | 69.8  [53.9, 82.8] | 79.7  [71.5, 86.4] | 81.6  [71.9, 89.1] | 75.0  [57.8, 87.9] |
| Bedtime home DBP | 87.4  [81.0, 92.3] | 88.9  [81.4, 94.1] | 83.7  [69.3, 93.2] | 88.6  [81.6, 93.6] | 90.8  [82.7, 95.9] | 83.3  [67.2, 93.6] |
| Office DBP | 90.4  [84.6, 94.5] | 95.5  [89.7, 98.5] | 78.3  [63.6, 89.1] | 90.6  [84.2, 95.1] | 95.5  [88.9, 98.8] | 79.5  [63.5, 90.7] |
| **Target: home SBP/DBP <125/75 mmHg; office SBP/DBP <130/80 mmHg** | | | | | | |
| Morning home SBP/DBP | 19.9  [13.8, 27.1] | 24.1  [16.4, 33.3] | 9.3  [2.6, 22.1] | 22.0  [15.0, 30.3] | 26.4  [17.6, 37.0] | 11.1  [3.1, 26.1] |
| Bedtime home SBP/DBP | 41.7  [33.8, 50.0] | 43.5  [34.0, 53.4] | 37.2  [23.0, 53.3] | 43.9  [35.0, 53.1] | 44.8  [34.1, 55.9] | 41.7  [25.5, 59.2] |
| Office SBP/DBP | 39.1  [31.4, 47.2] | 46.4  [36.8, 56.1] | 21.7  [10.9, 36.4] | 36.7  [28.4, 45.7] | 42.7  [32.3, 53.6] | 23.1  [11.1, 39.3] |
| **Target: home SBP <125 mmHg; office SBP <130 mmHg** | | | | | | |
| Morning home SBP | 41.1  [33.1, 49.3] | 42.6  [33.1, 52.5] | 37.2  [23.0, 53.3] | 39.8  [31.1, 49.1] | 40.2  [29.9, 51.3] | 38.9  [23.1, 56.5] |
| Bedtime home SBP | 55.0  [46.7, 63.1] | 55.6  [45.7, 65.1] | 53.5  [37.7, 68.8] | 56.9  [47.7, 65.8] | 56.3  [45.3, 66.9] | 58.3  [40.8, 74.5] |
| Office SBP | 46.8  [38.8, 54.9] | 52.7  [43.0, 62.3] | 32.6  [19.5, 48.0] | 44.5  [35.7, 53.6] | 49.4  [38.7, 60.2] | 33.3  [19.1, 50.2] |
| **Target: home DBP <75 mmHg; office DBP <80 mmHg** | | | | | | |
| Morning home DBP | 36.4  [28.8, 44.6] | 41.7  [32.3, 51.5] | 23.3  [11.8, 38.6] | 40.7  [31.9, 49.9] | 47.1  [36.3, 58.1] | 25.0  [12.1, 42.2] |
| Bedtime home DBP | 58.3  [50.0, 66.2] | 61.1  [51.3, 70.3] | 51.2  [35.5, 66.7] | 61.0  [51.8, 69.6] | 63.2  [52.2, 73.3] | 55.6  [38.1, 72.1] |
| Office DBP | 68.6  [60.7, 75.8] | 76.4  [67.3, 83.9] | 50.0  [34.9, 65.1] | 69.5  [60.8, 77.4] | 77.5  [67.4, 85.7] | 51.3  [34.8, 67.6] |

Data are achievement rates (%) and 95% confidence intervals.

Calculations of achievement rates for office BP used N = 156, N = 110, and N = 46 for overall, subgroups with and without T2DM, respectively in the FAS; in the PPS, N = 128, N = 89, and N = 39 respectively.

Abbreviations: DBP, diastolic blood pressure; FAS, full analysis set; PPS, per-protocol set; SBP, systolic blood pressure; T2DM, type 2 diabetes mellitus

**Supplementary Table 6.** Change in UACR and NT-proBNP from baseline to Week 12 (FAS and PPS)

|  | **Overall** | | **With T2DM** | | **Without T2DM** | | **eGFR_creat_**  **30 to <60 mL/min/1.73 m^2^** | | **eGFR_creat_**  **≥60 mL/min/1.73 m^2^** | |
| --- | --- | --- | --- | --- | --- | --- | --- | --- | --- | --- |
| **FAS** |  | |  | |  | |  | |  | |
| **UACR, mg/gCr** | **n** |  | **n** |  | **n** |  | **n** |  | **n** |  |
| Baseline | 175 | 280.3 ± 536.0  113.6  (47.9, 304.0) | 125 | 303.9 ± 578.7  150.2  (60.4, 332.9) | 50 | 221.5 ± 409.4  69.9  (39.9, 173.2) | 108 | 369.7 ± 652.1  177.1  (71.0, 472.2) | 59 | 127.1 ± 174.9  57.9  (37.9, 139.0) |
| Week 12 | 156 | 165.1 ± 388.9  55.3  (21.5, 178.2) | 110 | 166.9 ± 371.5  26.8  (64.0, 185.0) | 46 | 160.8 ± 432.0  16.96  (43.44, 141.74) | 94 | 225.4 ± 485.3  95.7  (35.2, 228.3) | 55 | 66.1 ± 84.6  31.4  (14.8, 67.6) |
| Change from baseline | 156 | −129.7 ± 319.2  −40.6  (−137.7, −17.1) | 110 | −156.4 ± 294.2  −58.9  (−212.0, −19.1) | 46 | −65.7 ± 368.1  −27.5  (−81.4, −11.1) | 94 | −177.6 ± 382.7  −70.1  (−221.6, −20.0) | 55 | −60.5 ± 129.4  −27.1  (−65.8, −13.0) |
| Percentage change in geometric mean from baseline [95% CI] |  | −55.2  [−61.2, −48.2]*** |  | −56.5  [−62.9, −48.9]*** |  | −52.0  [−65.1, −34.0]*** |  | −54.6  [−61.3, −46.7]*** |  | −55.4  [−63.9, −44.8]*** |
| **NT-proBNP, pg/mL** | **n** |  | **n** |  | **n** |  | **n** |  | **n** |  |
| Baseline | 170 | 133.3 ± 199.3  80.0  (37.0, 130.0) | 120 | 123.2 ± 184.0  80.0  (37.5, 129.5) | 50 | 157.5 ± 232.0  75.7  (37.0, 162.0) |  | Not calculated |  | Not calculated |
| Week 12 | 152 | 140.0 ± 276.3  61.8  (29.9, 139.0) | 107 | 142.3 ± 300.3  61.3  (31.3, 136.0) | 45 | 134.4 ± 211.4  62.3  (26.3, 144.0) |  | Not calculated |  | Not calculated |
| Change from baseline | 148 | −3.8 ± 190.7  −4.8  (−29.4, 6.0) | 103 | 9.3 ± 223.6  −2.4  (−21.0, 9.0) | 45 | −33.9 ± 65.8  −8.1  (−43.5, −0.6) |  | Not calculated |  | Not calculated |
| Percentage change in geometric mean from baseline [95% CI] |  | −14.1  [−20.4, −7.3]*** |  | −8.7  [−16.8, 0.1] |  | −25.3  [−34.4, −14.8]*** |  | Not calculated |  | Not calculated |
| **PPS** |  |  |  |  |  |  |  |  |  |  |
| **UACR, mg/gCr** | **n** |  | **n** |  | **n** |  | **n** |  | **n** |  |
| Baseline | 145 | 312.9 ± 580.3  136.5  (54.3, 358.0) | 102 | 345.6 ± 630.4  170.2  (66.7, 451.8) | 43 | 235.4 ± 435.9  72.4  (38.5, 207.2) | 103 | 381.0 ± 664.6  180.1  (72.6, 488.6) | 38 | 144.0 ± 210.7  48.0  (35.7, 139.0) |
| Week 12 | 128 | 182.1 ± 422.8  60.4  (22.5, 192.4) | 89 | 194.3 ± 407.8  88.2  (33.3, 203.9) | 39 | 154.4 ± 459.5  42.9  (13.9, 141.7) | 91 | 225.5 ± 491.9  92.0  (33.3, 228.3) | 34 | 74.7 ± 98.7  29.7  (12.4, 67.6) |
| Change from baseline | 128 | −148.4 ± 340.2  −57.5  (−183.3, −18.0) | 89 | −174.7 ± 315.9  −66.2  (−221.6, −20.0) | 39 | −88.4 ± 387.8  −29.0  (−87.7, −11.2) | 91 | −184.1 ± 387.2  −72.0  (−230.4, −20.6) | 34 | −68.3 ± 152.0  −27.3  (−62.7, −13.5) |
| Percentage change in geometric mean from baseline [95% CI] |  | −55.7  [−61.6, −48.9]*** |  | −54.5  [−60.9, −47.1]*** |  | −58.4  [−70.1, −42.2]*** |  | −56.1  [−62.5, −48.6]*** |  | −55.4  [−65.2, −43.0]*** |
| **NT-proBNP, pg/mL** | **n** |  | **n** |  | **n** |  | **n** |  | **n** |  |
| Baseline | 141 | 124.8 ± 183.9  80.4  (37.0, 126.0) | 98 | 126.1 ± 192.4  84.6  (37.0, 130.0) | 43 | 122.0 ± 165.1  68.7  (36.8, 125.0) |  | Not calculated |  | Not calculated |
| Week 12 | 127 | 124.6 ± 222.4  63.0  (30.0, 142.0) | 88 | 137.7 ± 255.3  64.5  (32.5, 145.0) | 39 | 95.0 ± 115.7  52.4  (25.0, 111.0) |  | Not calculated |  | Not calculated |
| Change from baseline | 123 | −12.5 ± 163.4  −4.0  (−29.0, 6.8) | 84 | −3.3 ± 191.9  −2.7  (−23.5, 11.0) | 39 | −32.1 ± 68.4  −7.5  (−42.0, 0.0) |  | Not calculated |  | Not calculated |
| Percentage change in geometric mean from baseline [95% CI] |  | −14.5  [−21.4, −7.0]*** |  | −9.2  [−18.0, 0.6] |  | −24.8  [−35.1, −12.9]*** |  | Not calculated |  | Not calculated |

Data are geometric mean ± standard deviation and median (Q1, Q3).

****P* <0.001 versus baseline, paired *t*-test.

*P-*values are only presented for percent change from baseline.

Abbreviations: CI, confidence interval; eGFR_creat_, creatinine-based estimated glomerular filtration rate; FAS, full analysis set; NT-proBNP, N-terminal pro-brain natriuretic peptide; PPS, per-protocol set; Q, quartile; T2DM, type 2 diabetes mellitus; UACR, urine albumin-to-creatinine ratio.

**Supplementary Table 7.** Exploratory endpoints: improvement, ≥30% reduction, and remission rates for UACR (FAS)^1^

|  | | **Overall (N = 156)** | | |
| --- | --- | --- | --- | --- |
|  | | **Sub-cohort at baseline** | | |
|  |  | A2 (n = 114) | A3 (n = 42) | A2 + A3 (n = 156) |
| Week 12 | A1^2^ | 47 (41.2) | 3 (7.1) | － |
|  | A2^3^ | － | 20 (47.6) | － |
|  | A3^4^ | 1 (0.9) | － | － |
|  | Improved | 47 (41.2) | 23 (54.8) | 70 (44.9) |
|  | Worsened | 1 (0.9) | － | － |
|  | ≥30% reduction | 79 (69.3) | 32 (76.2) | 111 (71.2) |
|  | Remission^5^ | 47 (41.2) | 3 (7.1) | 50 (32.1) |
|  | | **With T2DM (n = 110)** | | |
|  |  | **Sub-cohort at baseline** | | |
|  |  | A2 (n = 76) | A3 (n = 34) | A2 + A3 (n = 110) |
| Week 12 | A1^2^ | 29 (38.2) | 2 (5.9) | － |
|  | A2^3^ | － | 16 (47.1) | － |
|  | A3^4^ | 0 (0.0) | － | － |
|  | Improved | 29 (38.2) | 18 (52.9) | 47 (42.7) |
|  | Worsened | 0 (0.0) | － | － |
|  | ≥30% reduction | 52 (68.4) | 26 (76.5) | 78 (70.9) |
|  | Remission^5^ | 29 (38.2) | 2 (5.9) | 31 (28.2) |
|  | | **Without T2DM (n = 46)** | | |
|  |  | **Sub-cohort at baseline** | | |
|  |  | A2 (n = 38) | A3 (n = 8) | A2 + A3 (n = 46) |
| Week 12 | A1^2^ | 18 (47.4) | 1 (12.5) | － |
|  | A2^3^ | － | 4 (50.0) | － |
|  | A3^4^ | 1 (2.6) | － | － |
|  | Improved | 18 (47.4) | 5 (62.5) | 23 (50.0) |
|  | Worsened | 1 (2.6) | － | － |
|  | ≥30% reduction | 27 (71.1) | 6 (75.0) | 33 (71.7) |
|  | Remission^5^ | 18 (47.4) | 1 (12.5) | 19 (41.3) |
|  | | **eGFR_creat_** **30 to <60 mL/min/1.73 m^2^ (n = 94)** | | |
|  |  | **Sub-cohort at baseline** | | |
|  |  | A2 (n = 59) | A3 (n = 35) | A2 + A3 (n = 94) |
| Week 12 | A1^2^ | 20 (33.9) | 1 (2.9) | － |
|  | A2^3^ | － | 16 (45.7) | － |
|  | A3^4^ | 0 (0.0) | － | － |
|  | Improved | 20 (33.9) | 17 ( 48.6) | 37 (39.4) |
|  | Worsened | 0 (0.0) | － | － |
|  | ≥30% reduction | 39 (66.1) | 26 (74.3) | 65 (69.1) |
|  | Remission^5^ | 20 (33.9) | 1 (2.9) | 21 (22.3) |
|  | | **eGFR_creat_ ≥60 mL/min/1.73 m^2^ (n = 55)** | | |
|  |  | **Sub-cohort at baseline** | | |
|  |  | A2 (n = 49) | A3 (n = 6) | A2 + A3 (n = 55) |
| Week 12 | A1^2^ | 25 (51.0) | 1 (16.7) | － |
|  | A2^3^ | － | 4 (66.7) | － |
|  | A3^4^ | 0 (0.0) | － | － |
|  | Improved | 25 (51.0) | 5 (83.3) | 30 (54.5) |
|  | Worsened | 0 (0.0) | － | － |
|  | ≥30% reduction | 37 (75.5) | 5 (83.3) | 42 (76.4) |
|  | Remission^5^ | 25 (51.0) | 1 (16.7) | 26 (47.3) |

Data are n (%).

^1^Patients with missing data prior to administration or at week 12 were excluded.

^2^UACR <30 mg/gCr

^3^UACR 30 to <300 mg/gCr

^4^UACR 300 to <1000 mg/gCr

^5^Defined as transition to A1 combined with a ≥30% reduction in UACR from baseline

Abbreviations: eGFR_creat_, creatinine-based estimated glomerular filtration rate; FAS, full analysis set; T2DM, type 2 diabetes mellitus; UACR, urine albumin-to-creatinine ratio.

**Supplementary Table 8.** Exploratory endpoints: improvement, ≥30% reduction, and remission rates for UACR (PPS)^1^

|  | | **Overall (N = 128)** | | |
| --- | --- | --- | --- | --- |
|  | | **Sub-cohort at baseline** | | |
|  |  | A2 (n = 89) | A3 (n = 39) | A2 + A3 (n = 128) |
| Week 12 | A1^2^ | 38 (42.7) | 1 (2.6) | － |
|  | A2^3^ | － | 20 (51.3) | － |
|  | A3^4^ | 0 (0.0) | － | － |
|  | Improved | 38 (42.7) | 21 (53.8) | 59 (46.1) |
|  | Worsened | 0 (0.0) | － | － |
|  | ≥30% reduction | 62 (69.7) | 30 (76.9) | 92 (71.9) |
|  | Remission^5^ | 38 (42.7) | 1 (2.6) | 39 (30.5) |
|  | | **With T2DM (n = 89)** | | |
|  |  | **Sub-cohort at baseline** | | |
|  |  | A2 (n = 57) | A3 (n = 32) | A2 + A3 (n = 89) |
| Week 12 | A1^2^ | 21 (36.8) | 0 (0.0) | － |
|  | A2^3^ | － | 16 (50.0) | － |
|  | A3^4^ | 0 (0.0) | － | － |
|  | Improved | 21 (36.8) | 16 (50.0) | 37 (41.6) |
|  | Worsened | 0 (0.0) | － | － |
|  | ≥30% reduction | 38 (66.7) | 24 (75.0) | 62 (69.7) |
|  | Remission^5^ | 21 (36.8) | 0 (0.0) | 21 (23.6) |
|  | | **Without T2DM (n = 39)** | | |
|  |  | **Sub-cohort at baseline** | | |
|  |  | A2 (n = 32) | A3 (n = 7) | A2 + A3 (n = 39) |
| Week 12 | A1^2^ | 17 (53.1) | 1 (14.3) | － |
|  | A2^3^ | － | 4 (57.1) | － |
|  | A3^4^ | 0 (0.0) | － | － |
|  | Improved | 17 (53.1) | 5 (71.4) | 22 (56.4) |
|  | Worsened | 0 (0.0) | － |  |
|  | ≥30% reduction | 24 (75.0) | 6 (85.7) | 30 (76.9) |
|  | Remission^5^ | 17 (53.1) | 1 (14.3) | 18 (46.2) |
|  | | **eGFR_creat_ 30 to <60 mL/min/1.73 m^2^ (n = 91)** | | |
|  |  | **Sub-cohort at baseline** | | |
|  |  | A2 (n = 57) | A3 (n = 34) | A2 + A3 (n = 91) |
| Week 12 | A1^2^ | 20 (35.1) | 1 (2.9) | － |
|  | A2^3^ | － | 16 (47.1) | － |
|  | A3^4^ | 0 (0.0) | － | － |
|  | Improved | 20 (35.1) | 17 (50.0) | 37 (40.7) |
|  | Worsened | 0 (0.0) | － | － |
|  | ≥30% reduction | 38 (66.7) | 26 (76.5) | 64 (70.3) |
|  | Remission^5^ | 20 (35.1) | 1 (2.9) | 21 (23.1) |
|  | | **eGFR_creat_ ≥60 mL/min/1.73 m^2^ (n = 34)** | | |
|  |  | **Sub-cohort at baseline** | | |
|  |  | A2 (n = 29) | A3 (n = 5) | A2 + A3 (n = 34) |
| Week 12 | A1^2^ | 17 (58.6) | 0 (0.0) | － |
|  | A2^3^ | － | 4 (80.0) | － |
|  | A3^4^ | 0 (0.0) | － | － |
|  | Improved | 17 (58.6) | 4 (80.0) | 21 (61.8) |
|  | Worsened | 0 (0.0) | － | － |
|  | ≥30% reduction | 23 (79.3) | 4 (80.0) | 27 (79.4) |
|  | Remission^5^ | 17 (58.6) | 0 (0.0) | 17 (50.0) |

Data are n (%).

^1^Patients with missing data prior to administration or at week 12 were excluded.

^2^UACR <30 mg/gCr

^3^UACR 30 to <300 mg/gCr

^4^UACR 300 to <1000 mg/gCr

^5^Defined as transition to A1 combined with a ≥30% reduction in UACR from baseline

Abbreviations: eGFR_creat_, creatinine-based estimated glomerular filtration rate; PPS, per-protocol set; T2DM, type 2 diabetes mellitus; UACR, urine albumin-to-creatinine ratio.

**Supplementary Table 9.** Change in serum potassium and eGFR_creat_ from baseline to Week 12 (safety analysis set)

|  | **Overall** | | **With T2DM** | | **Without T2DM** | | **eGFR_creat_ 30 to <60 mL/min/1.73 m^2^** | | **eGFR_creat_ ≥60**  **mL/min/1.73 m^2^** | |
| --- | --- | --- | --- | --- | --- | --- | --- | --- | --- | --- |
|  | **n** | **Mean ± SD** | **n** | **Mean ± SD** | **n** | **Mean ± SD** | **n** | **Mean ± SD** | **n** | **Mean ± SD** |
| **Serum potassium, mEq/L** |  |  |  |  |  |  |  |  |  |  |
| Baseline | 172 | 4.2 ± 0.4 | 126 | 4.2 ± 0.4 | 46 | 4.1 ± 0.5 | 110 | 4.3 ± 0.4 | 62 | 4.1 ± 0.5 |
| Week 2 | 173 | 4.4 ± 0.4 | 120 | 4.5 ± 0.4 | 53 | 4.3 ± 0.5 | 105 | 4.5 ± 0.4 | 60 | 4.3 ± 0.4 |
| Change from baseline | 165 | 0.3 ± 0.3 | 119 | 0.3 ± 0.3 | 46 | 0.3 ± 0.3 | 105 | 0.3 ± 0.3 | 60 | 0.2 ± 0.3 |
| Week 4 | 165 | 4.4 ± 0.4 | 115 | 4.4 ± 0.4 | 50 | 4.4 ± 0.5 | 102 | 4.4 ± 0.4 | 56 | 4.3 ± 0.4 |
| Change from baseline | 158 | 0.2 ± 0.4 | 114 | 0.2 ± 0.4 | 44 | 0.3 ± 0.3 | 102 | 0.2 ± 0.4 | 56 | 0.2 ± 0.4 |
| Week 8 | 146 | 4.4 ± 0.4 | 109 | 4.4 ± 0.4 | 37 | 4.5 ± 0.5 | 95 | 4.5 ± 0.4 | 48 | 4.3 ± 0.4 |
| Change from baseline | 143 | 0.3 ± 0.4 | 108 | 0.2 ± 0.4 | 35 | 0.4 ± 0.4 | 95 | 0.3 ± 0.4 | 48 | 0.3 ± 0.3 |
| Week 12 | 158 | 4.4 ± 0.4 | 110 | 4.5 ± 0.4 | 48 | 4.4 ± 0.5 | 96 | 4.5 ± 0.4 | 55 | 4.3 ± 0.5 |
| Change from baseline | 151 | 0.3 ± 0.4 | 109 | 0.3 ± 0.4 | 42 | 0.3 ± 0.4 | 96 | 0.3 ± 0.4 | 55 | 0.2 ± 0.4 |
| **eGFR_creat_, mL/min/1.73 m^2^** |  |  |  |  |  |  |  |  |  |  |
| Baseline | 172 | 60.5 ± 18.1 | 126 | 57.4 ± 16.2 | 46 | 68.9 ± 20.3 | 110 | 49.8 ± 7.6 | 62 | 79.5 ± 15.4 |
| Week 2 | 173 | 58.0 ± 18.3 | 120 | 54.8 ± 16.9 | 53 | 65.3 ± 19.5 | 105 | 47.2 ± 8.4 | 60 | 74.5 ± 17.0 |
| Change from baseline | 165 | −3.8 ± 7.0 | 119 | −3.2 ± 6.7 | 46 | −5.3 ± 7.4 | 105 | −2.7 ± 5.0 | 60 | −5.5 ± 9.3 |
| Week 4 | 165 | 56.6 ± 17.7 | 115 | 54.1 ± 17.0 | 50 | 62.4 ± 18.1 | 102 | 46.6 ± 9.0 | 56 | 72.9 ± 16.1 |
| Change from baseline | 158 | −4.4 ± 7.0 | 114 | −3.6 ± 5.9 | 44 | −6.6 ± 8.9 | 102 | −3.3 ± 5.2 | 56 | −6.5 ± 9.0 |
| Week 8 | 146 | 55.0 ± 17.3 | 109 | 52.9 ± 15.4 | 37 | 61.1 ± 20.8 | 95 | 46.2 ± 8.5 | 48 | 71.4 ± 17.1 |
| Change from baseline | 143 | −4.7 ± 6.8 | 108 | −4.2 ± 6.4 | 35 | −6.6 ± 7.7 | 95 | −3.5 ± 4.8 | 48 | −7.2 ± 9.3 |
| Week 12 | 157 | 56.4 ± 18.4 | 110 | 53.6 ± 16.8 | 47 | 63.1 ± 20.3 | 95 | 45.6 ± 8.8 | 55 | 73.8 ± 17.5 |
| Change from baseline | 150 | −4.9 ± 7.9 | 109 | −4.4 ± 6.7 | 41 | −6.2 ± 10.5 | 95 | −4.3 ± 6.3 | 55 | −5.9 ± 10.2 |

*P*-values were not calculated.

Abbreviations: eGFR_creat_, creatinine-based estimated glomerular filtration rate; SD, standard deviation; T2DM, type 2 diabetes mellitus.

**Supplemental Table 10.** Incidence of serum potassium level ≥5.5 mEq/L (safety analysis set)

|  | **Overall**  **(N = 180)** | **With T2DM**  **(n = 127)** | **Without T2DM**  **(n = 53)** | **eGFR_creat_ 30 to <60**  **mL/min/1.73 m^2^**  **(n = 110)** | **eGFR_creat_ ≥60**  **mL/min/1.73 m^2^**  **(n = 62)** |
| --- | --- | --- | --- | --- | --- |
| ≥5.5 mEq/L | 10 (5.6) | 4 (3.1) | 6 (11.3) | 6 (5.5) | 2 (3.2) |

Data are n (%).

Abbreviations: eGFR_creat_, creatinine-based estimated glomerular filtration rate; T2DM, type 2 diabetes mellitus.
